# Reducing *Campylobacter jejuni* Colonization of Poultry via Vaccination

**DOI:** 10.1371/journal.pone.0114254

**Published:** 2014-12-04

**Authors:** Jason M. Neal-McKinney, Derrick R. Samuelson, Tyson P. Eucker, Mark S. Nissen, Rocio Crespo, Michael E. Konkel

**Affiliations:** 1 School of Molecular Biosciences, College of Veterinary Medicine, Washington State University, Pullman, Washington, United States of America; 2 Avian Health & Food Safety Laboratory, College of Veterinary Medicine, Washington State University, Puyallup, Washington, United States of America; Charité-University Medicine Berlin, Germany

## Abstract

*Campylobacter jejuni* is a leading bacterial cause of human gastrointestinal disease worldwide. While *C. jejuni* is a commensal organism in chickens, case-studies have demonstrated a link between infection with *C. jejuni* and the consumption of foods that have been cross-contaminated with raw or undercooked poultry. We hypothesized that vaccination of chickens with *C. jejuni* surface-exposed colonization proteins (SECPs) would reduce the ability of *C. jejuni* to colonize chickens, thereby reducing the contamination of poultry products at the retail level and potentially providing a safer food product for consumers. To test our hypothesis, we injected chickens with recombinant *C. jejuni* peptides from CadF, FlaA, FlpA, CmeC, and a CadF-FlaA-FlpA fusion protein. Seven days following challenge, chickens were necropsied and cecal contents were serially diluted and plated to determine the number of *C. jejuni* per gram of material. The sera from the chickens were also analyzed to determine the concentration and specificity of antibodies reactive against the *C. jejuni* SECPs. Vaccination of chickens with the CadF, FlaA, and FlpA peptides resulted in a reduction in the number of *C. jejuni* in the ceca compared to the non-vaccinated *C. jejuni*-challenged group. The greatest reduction in *C. jejuni* colonization was observed in chickens injected with the FlaA, FlpA, or CadF-FlaA-FlpA fusion proteins. Vaccination of chickens with different SECPs resulted in the production of *C. jejuni*-specific IgY antibodies. In summary, we show that the vaccination of poultry with individual *C. jejuni* SECPs or a combination of SECPs provides protection of chickens from *C. jejuni* colonization.

## Introduction

*Campylobacter* species are the most common culture-proven cause of bacterial gastroenteritis worldwide, accounting for 400–500 million cases of diarrhea each year [1]. In the United States, the annual incidence of infection with *C. jejuni* (*Campylobacter*iosis) is estimated to be between 2.4 to 4 million cases [2]. In 2013, the incidence of *Campylobacter*iosis was 13.82 culture confirmed cases/100,000 persons in the United States, although the actual number of infections is likely higher (http://www.cdc.gov/foodnet/data/trends/tables/2013/table2a-b.html#table-2b). *C. jejuni* infection is also the most common zoonosis in the European Union, and a significant increasing trend has been observed in the five years from 2008–2012 [3]. In 2012, the EU notification rate was 55.49 cases/100,000 persons [4]. In addition to acute gastroenteritis, infection with particular strains of *C. jejuni* correlates with a higher incidence of Guillain-Barré syndrome (GBS). GBS, an autoimmune disease affecting the peripheral nervous system, is the leading cause of flaccid paralysis in the post-polio era [5]. The current cost associated with treating acute *C. jejuni* infections and GBS is estimated to be $1.2 billion per year in the U.S and 2.4 Billion € in the EU. [3], [6], [7].

*Campylobacter jejuni* colonizes chickens at densities of 10^8^ colony forming units (CFU)/gram of cecal contents or greater without causing disease [8], [9]. After *C. jejuni* colonizes a few birds in a flock, it rapidly spreads throughout the flock and the bacteria remain present throughout the bird’s lifespan [9], [10]. Strikingly, up to 90% of domestic chicken carcasses are contaminated with *C. jejuni* at the time of sale, depending on source and seasonal variations [11], [12]. Methods are currently being developed to reduce the burden of *C. jejuni* at all stages of production [4], [13], [14], [15], [16], [17], [18], [19], including measures to prevent chicken exposure to *C. jejuni* [14], [15], [16], [20], [21], treatments to reduce the load of *C. jejuni* within birds [22], [23], [24], [25], [26], [27], [28], strategies to reduce contamination during slaughter [29], [30], [31], [32], and processes to remove/kill *C. jejuni* from the surface of meat products [33], [34], [35], [36]. Quantitative risk assessment indicates that a 3 log_10_ reduction of *C. jejuni* in the intestines of chickens or a 2 log_10_ reduction on the carcass would reduce the public health risk 90% [4], [37].

Strategies to reduce the carriage of *C. jejuni* within poultry include: 1) the administration of compounds with anti-*C. jejuni* activity [24], [26], [28], [38], [39]; 2) the use of probiotic bacteria that compete with *C. jejuni* for colonization or produce inhibitory metabolites [25], [27], [40], [41]; 3) the application of bacteriophage specific to *C. jejuni* [22],[42],[43],[44]; and 4) the vaccination of chickens with *C. jejuni* antigens [23], [45], [46], [47]. In this study, we will describe a strategy to reduce colonization of poultry via vaccination with *C. jejuni* peptides.

Various vaccination strategies are currently being developed to combat *C. jejuni* in poultry. Oral vaccination with whole-killed *C. jejuni* resulted in a moderate decline in colonization [47], whereas oral vaccination with recombinant CmeC has been shown to induce a serum antibody response, but did not confer protection to *C. jejuni* colonization. Several studies have shown the efficacy of using live attenuated *Salmonella* to deliver the CjaA [23], [46], [48], [49] CjaD [46] or Dps [45] antigens to poultry. Attenuated *Eimeria* parasites are another alternative delivery platform that have been used to deliver CjaA antigen [50]. Annamalai et al. successfully demonstrated a reduction in poultry colonization by vaccination with nano-particle encapsulated *C. jejuni* outer-membrane proteins [51] The administration of egg-derived IgY antibodies specific to *C. jejuni* as a passive immunotherapy strategy is also currently being investigated [52]. Additional studies are needed to identify the antigens and delivery methods that are most effective in inhibiting *C. jejuni* colonization.

Critical advances in our understanding of *C. jejuni* colonization of chickens have occurred in the past few years. It is now known that the level of maternal antibodies in chicks remains high for 3 to 4 days after hatching and then gradually decreases to undetectable levels by ∼2 to 3 weeks of age [9]. Accordingly, *C. jejuni* colonization of chickens coincides with the decrease (absence) in antibodies reactive against the bacterium. Researchers have also found that the production of anti-*C. jejuni* antibodies by the chicken prior to exposure results in a decrease in the bacterium’s ability to colonize chickens [47], [49]. We have identified *C. jejuni* proteins that generate protective maternal antibodies in hens [53]. More specifically, we have detected antibodies directed against *C. jejuni* adhesins (*i.e.,* CadF and FlpA), flagellar proteins (*i.e.,* FlaA and FlaB), and other membrane associated proteins in the sera of newly hatched birds. Thus, it may be possible to reduce and/or prevent *C. jejuni* colonization of chickens by inducing the production of *Campylobacter*-specific antibodies.

Significant progress has been made in identifying the *C. jejuni* proteins that promote colonization of chickens. We will refer to the surface-exposed *C. jejuni* proteins that contribute in the colonization of poultry as *C. jejuni* surface-exposed colonization proteins (SECPs). These proteins can be subdivided into groups of proteins that play a role in host cell adherence (adhesins), motility, or survival within the host intestine. The *C. jejuni* adhesins, which are synthesized constitutively by *C. jejuni* [54], include CadF [*Campylobacter* adhesion to fibronectin (Fn)] [55], [56], FlpA (Fibronectin-like protein A) [57], [58], and CapA (*Campylobacter* adhesion protein A) [57], [59]. In addition to adhesins, bacterial motility plays an important role in the colonization of animals [60], [61], [62], [63]. Maximal *C. jejuni* motility requires the FlaA (major) and FlaB (minor) filament proteins. Finally, there are other membrane-associated proteins, such as CmeC and the major outer membrane protein (PorA), that may serve a function in bacterial survival in the intestines of chickens [64], [65].

The proteins (CadF, FlpA, FlaA, and CmeC) used in this study were selected based on one or more of the following criteria: 1) the protein is known or proposed to be involved in *C. jejuni* colonization of chickens (*e.g.*, is necessary for bacterial motility, cell adherence, nutrient acquisition); 2) the protein contains putative surface exposed epitopes, so that antibodies against the protein may bind to the surface of the bacterium and prevent an essential step in colonization (*e.g.*, binding of the bacterium to a cell lining the intestinal tract, motility, etc.); and 3) the protein stimulates an immune response in the context of natural colonization, as demonstrated by the production of maternal antibodies. Based on previous studies, the CadF and FlpA proteins were proposed to have the greatest potential for reducing *C. jejuni* colonization of chickens [57], [66]. These two fibronectin-binding proteins fulfill the first three criteria outlined above and are known to promote the binding of *C. jejuni* to cultured epithelial cells. FlaA was also considered a high priority target, as it also fulfills the three criteria. The filament of the *C. jejuni* flagellum is composed of two proteins termed FlaA and FlaB. FlaA is the major constituent of the flagellar filament and is required for bacterial motility [67], [68], [69]. CmeC encodes for one component of a multidrug efflux system (CmeABC). The CmeABC efflux system has been shown to play a role in resistance to deoxycholic acid and in permitting chicken colonization [70].

The goal of this study was to compare the efficacy of vaccination with *C. jejuni* SECPs in reducing the load of *C. jejuni* in the digestive tract of chickens. We chose to target the CadF, FlpA, FlaA, and CmeC proteins, as they are required for maximal chicken colonization, well-conserved amongst *C. jejuni* strains, and present on the surface of the bacterium. Our central hypothesis is that reducing the total number of *C. jejuni* in the intestines of poultry will reduce the risk of human infection.

## Materials and Methods

### Bacterial strains and growth

The bacterial strains used in this study are listed in Table S1. *Campylobacter jejuni* F38011 was cultured on Mueller-Hinton agar plates supplemented with 5% bovine blood (MH-blood agar) under microaerobic conditions (5% O_2_, 10% CO_2_, 85% N_2_). The *C. jejuni* were passaged to fresh MH-blood agar plates every 24 to 48 hr. *Escherichia coli* XL-1 Blue (Stratagene, Garden Grove, CA) and BL21DE3 (Novagen, Madison, WI) were maintained on Luria-Bertani (LB) agar plates or in LB broth aerobically at 37°C.

### Construction and verification of recombinant vectors

The primer sets used to clone the DNA fragments encoding each recombinant peptide (CadF, FlaA, FlpA, and CmeC, and the CadF-FlaA-FlpA fusion protein) are listed in Table S2. The fragments selected for the expression of CadF, FlaA, FlpA, and CmeC GST tagged proteins are 90 amino acids in length. The CadF-FlaA-FlpA fusion protein, which is comprised of 30 residues from each SECP (*i.e.,* CadF, FlaA, and FlpA), will be referred to as the ‘trifecta’ from this point forward. The trifecta epitope sequence was synthesized by Integrated DNA Technologies (San Diego, CA, USA) and subcloned into vectors for expression and synthesis. The fragments selected for the expression of CadF, FlaA, FlpA, and CmeC His tagged proteins are full-length sequences minus the signal peptide. Detailed descriptions of the residues selected from each SECP are indicated in Table S3.

### Expression and purification of the *C. jejuni* SECPs

*E. coli* strains harboring pGEX-5X-1 (GE Healthcare, Buckinghamshire, UK) were grown on media supplemented with 100 µg/ml ampicillin. Strains that harbor the pET-24b plasmid (Qiagen, Valencia, CA) were grown on media supplemented with 50 µg/ml of kanamycin. Construction and expression of the recombinant N-terminal glutathione S-transferase (GST)-tagged and the recombinant C-terminal hexa-histidine (6X HIS)-tagged proteins were performed using standard molecular biology techniques described previously [58]. Purification of GST and His fusion proteins were performed utilizing glutathione spin columns and HisPur Ni-NTA spin columns (Pierce, Rockford, IL), respectively, according to the manufacturer’s instructions.

### Broiler chicks

Two-day old specific-pathogen free chicks (Cornish cross broilers) were received from the Avian Health & Food Safety Laboratory in Puyallup, WA, and placed into isolation chambers containing mesh wire on the floor of the cages. The chickens were given water and commercial chick starter feed *ad libitum*. All animal work was performed using protocols approved by the Institutional Animal Care and Use Committee (IACUC, protocol no. 4026) at Washington State University. All efforts were made to raise and euthanize the animals humanely.

### Vaccination of chickens

Peptides used for vaccinations are described in Tables S3 and S4. Seventy-seven chicks were subdivided into seven groups. At 6 days of age, the chicks were injected with 240 µg of the GST-tagged 90 mer peptides. At 16 days of age, a booster injection was given with 240 µg of full-length CadF-His, FlaA-His, FlpA-His, and CmeC-His, or a mixture of 80 µg CadF-His, 80 µg FlaA-His, and 80 µg FlpA-His (trifecta group). All peptides (antigens) were emulsified in Montanide ISA 70 VG (Seppic, Paris, France), a commonly used adjuvant for poultry vaccines [71], [72], at a ratio of 30% antigen and 70% adjuvant. For the primary and booster injections, 200 µL of peptide/adjuvant mixture was injected into the left and right breast muscle. Two groups were not vaccinated, with one group serving as the uninfected negative control chicks, and the other as the positive control chicks infected with *C. jejuni* but not vaccinated. Results of a pilot experiment were consistent with the results presented herein (Figures S1 and S2).

### *C. jejuni* challenge experiments

Chicks were challenged with 2×10^8^ colony-forming units (CFU) of *C. jejuni* orally at 20 days of age. The birds were humanely euthanized by carbon dioxide asphyxiation and necropsied at day 27. One cecum from each bird was weighed and stomached in an equal volume of MH broth (1 mL broth per gram of cecal contents). The cecal contents were then serially diluted and plated on Campy-Cefex Agar for enumeration. Blood was aspirated directly from the heart of necropsied chickens to obtain serum for antibody analysis.

### Enzyme-linked immunosorbent assay (ELISA)

*C. jejuni* whole cell lysates (WCLs) were generated by sonication. Ninety-six well ELISA plates were purchased from Costar (Part No. 3797). Wells were coated with 1 µg of WCL in bicarbonate buffer (pH 9.5) overnight at 4°C. The wells were then blocked with 5% ovalbumin (Sigma-A5503; Sigma-Aldrich, St. Louis, MO) for one hour at RT. Chicken serum (100 µl) from each individual bird was diluted 1∶10 in PBS containing 5% ovalbumin and added to wells in duplicate and incubated for 1 hour at RT. The wells were then probed with rabbit anti-chicken IgY (Sigma A9046) at a dilution of 1∶1000 in PBS with 5% ovalbumin. Horseradish peroxidase (HRP) conjugated goat anti-rabbit IgG (Sigma-A6154) was then added for 1 hour at RT at a dilution of 1∶5000 in PBS with 5% ovalbumin. Tetramethylbenzidine solution (TMB, Thermo Scientific, Rockford, IL) was then added to the each well and developed for 15 minutes at RT prior to stopping the reaction with 2 M H_2_SO_4_. ELISA plates were read at absorbance 450 (A_450_) using an EL_x_808 microplate reader (BioTek Instruments Inc., Winooski, VT).

### Outer-membrane protein extraction

Outer-membrane proteins were extracted from the *C. jejuni* F38011 wild-type strain as described previously [56].

### Immunoblot analysis

Sera from the CadF, FlaA, FlpA, and trifecta injected groups were analyzed by immunoblot to determine the specificity of antibody reactivity. Whole-cell lysates of the *C. jejuni* wild-type strain, *cadF flpA* mutant, and *flaA flaB* mutant were prepared by lysis in SDS-PAGE Sample Buffer and normalized using the bicinchoninic acid assay. Whole-cell lysates and outer-membrane protein extracts were separated by SDS-PAGE and transferred to polyvinylidene fluoride (PVDF) membranes. The membranes were blocked for 30 min with 9% non-fat dried milk (NFDM) in Tris-buffered saline buffer containing 1% Tween 20 (TBST buffer), washed three times in TBST (five min per wash), and probed overnight with chick sera diluted 1∶1000 in TBST containing 9% NFDM. The PVDF membranes then were washed three times with TBST buffer, and probed for 1 hour with anti-chicken IgY conjugated to horseradish peroxidase at a dilution of 1∶2000 in TBST. After 4 washes in TBST, the blots were developed using Western Lightning ECL reagent (Perkin Elmer, Waltham, MA), and imaged using a LAS 4000 mini multi-imager (GE Healthcare).

### Statistical analysis

Graphing and statistical analysis was performed using Prism 6.0 (GraphPad). 95% Confidence Intervals were calculated based on the serum reactivity of the untreated control group and non-vaccinated, *C. jejuni*-challenged group, using Microsoft Excel. Serum absorbance values above the 95% confidence interval are considered to be reactive against *C. jejuni.*

## Results

### Antigen selection and validation of *C. jejuni* recombinant proteins

**Figure 1:**
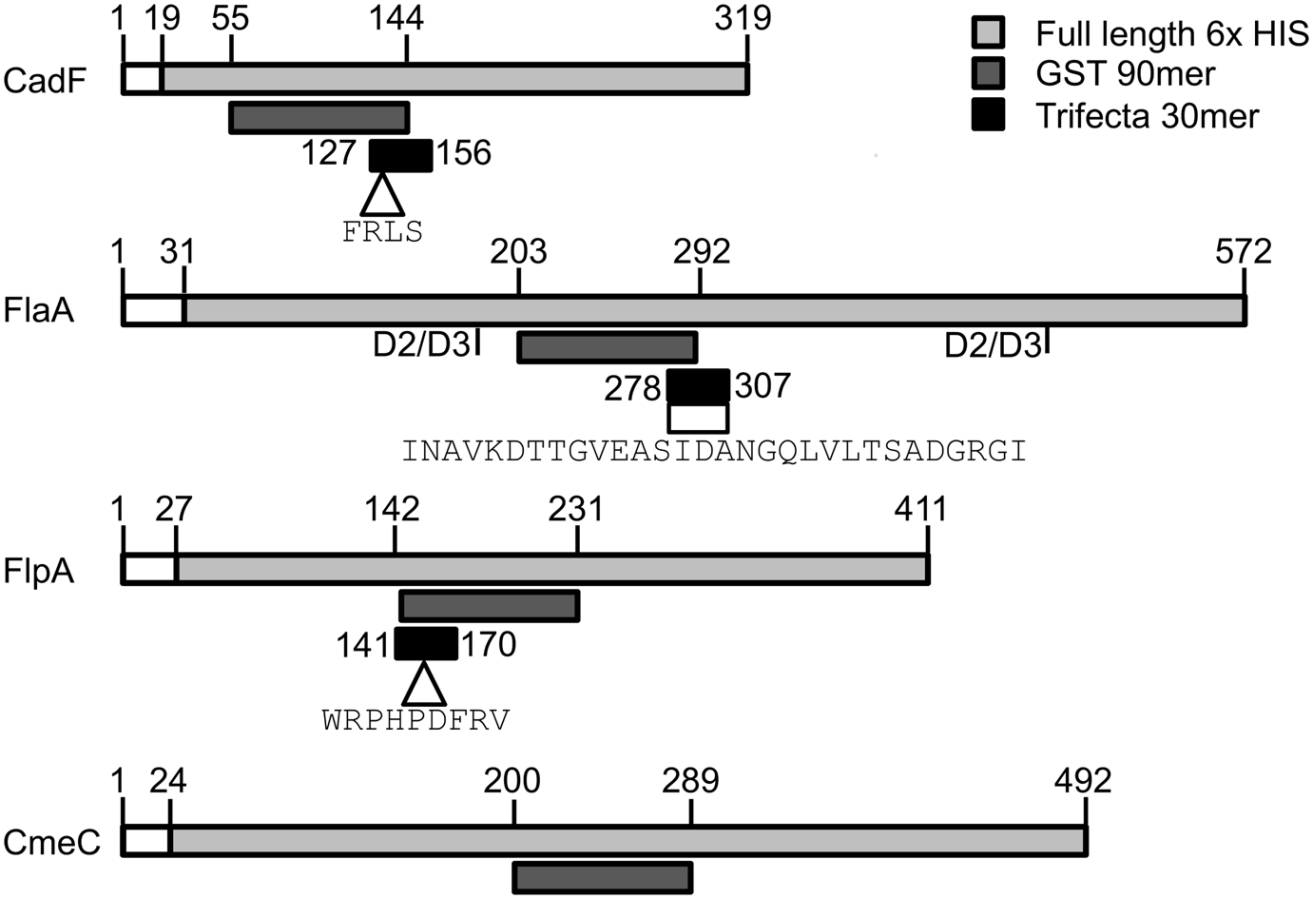
Domains of the *C. jejuni* proteins targeted for vaccination. The residues of CadF, FlaA, FlpA, and CmeC used for vaccination of chicks are shown above the diagram for each peptide. The portions used for the 6X His-tagged peptides are shown in light gray, the GST-tagged 90 mer fragments are shown in dark-gray, and the 30 mer peptides used to create the trifecta peptide are shown in black. The signal peptides (CadF, FlpA, and CmeC) or sequences required for Type 3 secretion (FlaA) were excluded from the 6X His-tagged peptides, and are shown in white. The arrows under the diagrams for CadF and FlpA indicate residues determined to be required for adherence to fibronectin. The box under the FlaA diagram represents the area chosen to create a synthetic FlaA 30 mer peptide based on the consensus sequence shown below. This region falls within the borders of the D2 and D3 domains of FlaA, which are predicted to be surface-exposed in the native protein. The 90 mer region chosen for CmeC falls within a hydrophilic region of the protein that is predicted to be surface-exposed.

The rationale for this experimental design was to narrow the site of interest within a *C. jejuni* SECP to 30 residues and to test if a combination of fragments from different proteins would generate enhanced protection (*i.e.*, a reduction in *C. jejuni* colonization) versus injection with an individual *C. jejuni* SECP. We defined the ‘site of interest’ for the CadF, FlaA, and FlpA proteins to be the site most likely to be surface exposed, immunogenic, and protective (*i.e.,* required for protein function). Our strategy also involved generating both GST-tagged 90 mers and His-tagged full-length proteins (minus the signal peptide) for CadF, FlaA, FlpA, and CmeC (Figure 1). The trifecta (CadF-FlaA-FlpA) peptide, composed of three 30 mers, was generated only as a GST-tagged protein. The rationale for using two distinct peptides is to generate an antibody response against a specific region of the protein using the first injection with the GST-90 mers. The booster injection then used a full-length version of the peptide to enhance the immune response without including the 26 kDa GST tag, resulting in specificity against the 90 mer fragment (and not GST). The 90 mer regions used were chosen based on putative surface exposure (*i.e.,* hydrophilicity predictions), conservation amongst *C. jejuni* strains, and the presence of sequences required for protein function.

Noteworthy is that the residues that comprise the CadF and FlpA 30 mers are identical to those predicted to be encoded from *C. jejuni* NCTC 11168. In contrast, the residues that comprise the FlaA 30 mer represent a consensus sequence derived from multiple *C. jejuni* strains. Figure 1 shows the consensus sequence of the FlaA protein from twenty-four *C. jejuni* strains and two *C. coli* strains. The FlaA sequences used in this alignment are listed in Table S5. Importantly, the FlaA consensus sequence contains linear epitopes present in every *C. jejuni* FlaA sequence examined.

**Figure 2:**
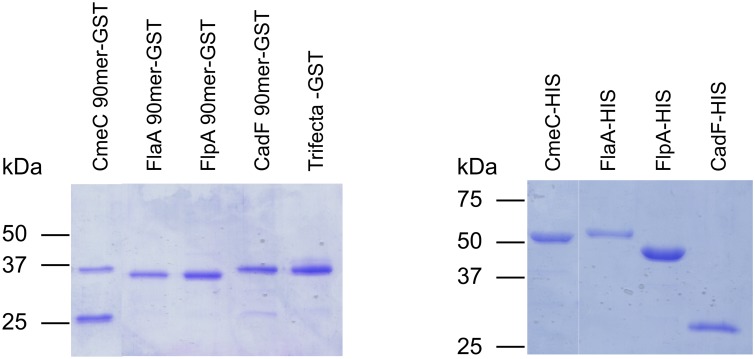
Purified GST- and 6X His-tagged proteins. Purified protein extracts were separated in SDS–12.5% polyacrylamide gels and stained with Coomassie Brilliant Blue R-250. The molecular mass of the protein standards are listed in kDa.

SDS-PAGE analysis of the purified GST-tagged 90 mers and His-tagged full-length proteins (minus the signal peptide) for CadF, FlaA, FlpA, and CmeC is shown in Figure 2. Also shown in Figure 2 is the purified GST-tagged CadF-FlaA-FlpA trifecta protein. The *M*_r_ of the recombinant proteins is similar to the calculated molecular masses (Table S4). The CadF 90 mer peptide contains the FRLS domain, which is required for adherence to fibronectin [55]. The FlaA 90 mer peptide was chosen based on putative surface exposure, as it is located within the D2 and D3 domains of flagellin. The FlpA 90 mer peptide contains a Type III repeat found within fibronectin, as well as the residues WRPHPDFRV, which are required for binding to fibronectin [73]. The CmeC 90 mer peptide is predicted to be exposed to the extracellular environment, based on the hydrophilicity profile. The CadF-FlaA-FlpA trifecta was comprised of 30 residues from each SECP (Table S3).

### Protective efficacy of *C. jejuni* recombinant proteins

**Figure 3:**
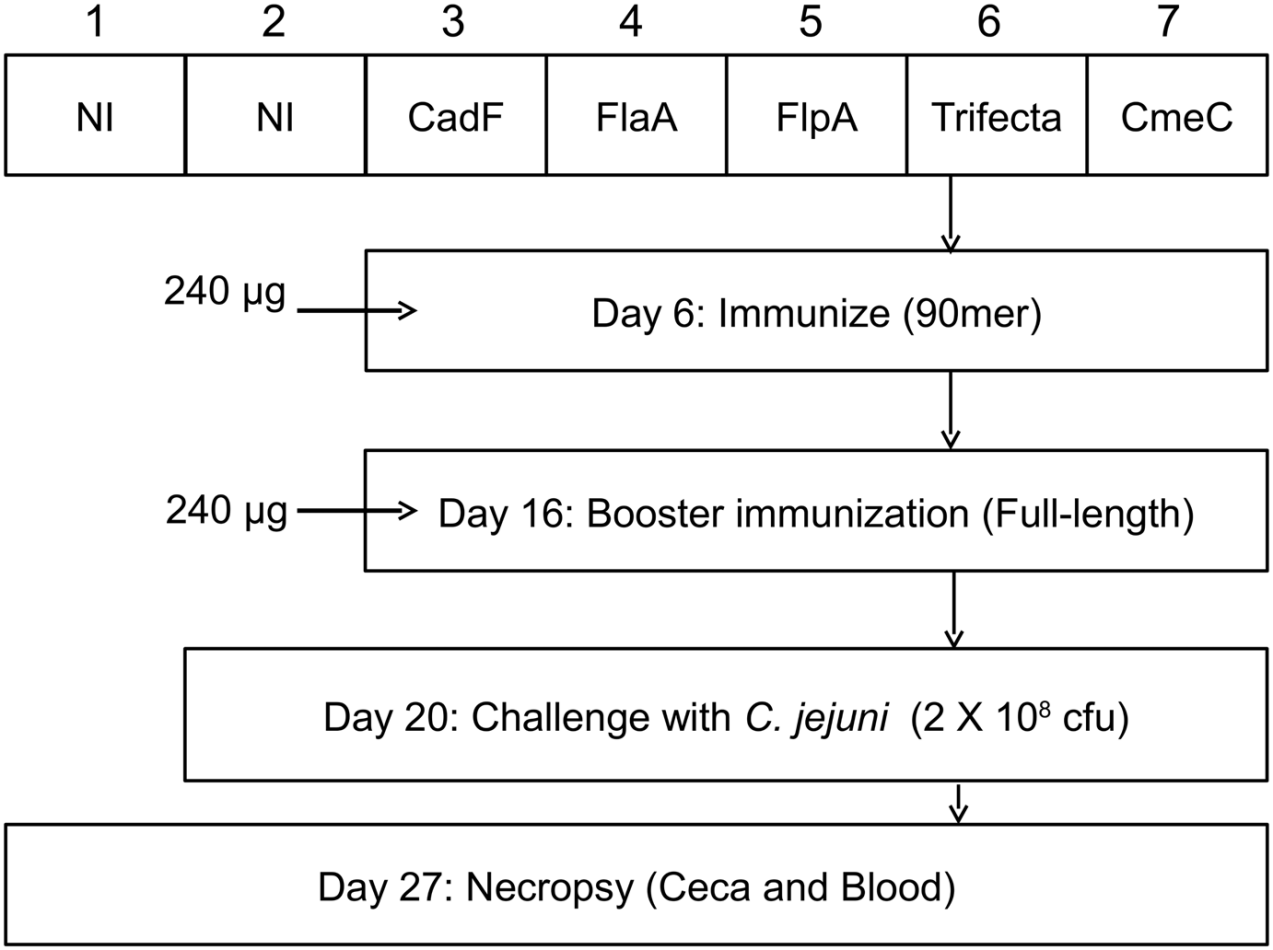
Experimental design of chicken vaccination experiment. The first peptide injections were given at a dose of 240 µg (GST-CadF, GST-FlaA, GST-FlpA, GST-CmeC, and the CadF-FlaA-FlpA GST-trifecta groups) and the booster injections were given at a dose of 240 µg (CadF-His, FlpA-His, GST-FlaA, GST-CmeC, and CadF-FlaA-FlpA GST-trifecta). Groups 1 and 2 were not injected with a *C. jejuni* SECP (NI = no injection). Each chicken within Groups 2 through 7 received a dose of 2×10^8^ CFU of *C. jejuni* at day 20. The chickens were necropsied at 27 days of age.

The experimental design of the chicken vaccination experiment is shown in Figure 3. One-day-old chicks were divided into seven groups and placed in isolator chambers. At six days of age, the chicks were injected with 240 µg of the GST-tagged 90 mer peptides GST-CadF, GST-FlaA, GST-FlpA, GST-CmeC, and GST-trifecta. The booster injection given on day 16 consisted of 240 µg of full-length CadF-His, FlaA-His, FlpA-His, and CmeC-His, or 240 µg of a mixture of 80 µg each of CadF-His, FlaA-His, and FlpA-His. Two groups of non-injected birds were included as controls; one group was not challenged while the other was challenged with *C. jejuni*. At 20 days of age, the chicks were orally challenged with 2×10^8^ CFU of *C. jejuni* F38011 wild-type strain. One week later (day 27) the chicks were necropsied and ceca and blood collected for analysis.

**Figure 4:**
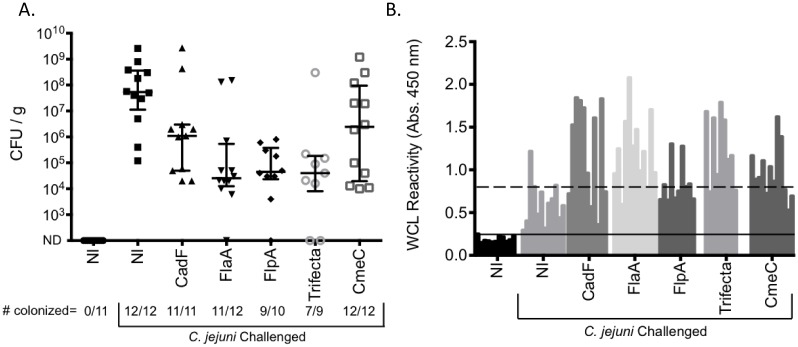
Vaccination with CadF, FlaA, FlpA, and trifecta peptides reduces *C. jejuni* colonization. **Panel A:** The number of CFU of *C. jejuni* was determined by serially plating diluted cecal contents on Campy-Cefex plates. The median level of colonization and interquartile range are shown for each group. The number of birds colonized in each group is shown below the graph. NI = no injection. ND = not detected; limit of detection is 10^3^ CFU/gram of cecal contents. **Panel B:** Sera was isolated from blood collected from the chickens at the time of necropsy and examined via ELISA using microtiter plates coated with a *C. jejuni* whole-cell lysate. Chicken IgY bound to the coated wells was detected with a rabbit anti-chicken IgY antibody conjugated to HRP. 95% confidence intervals were calculated based on the level of sera reactivity in the non-vaccinated, unchallenged group (solid line) and non-vaccinated, non-challenged groups of chickens (dashed line).

The median level of colonization in the *C. jejuni*-treated, non-vaccinated control group was 5.35×10^7^ CFU/gram (Figure 4, Panel A). The trifecta vaccination resulted in a significant reduction in *C. jejuni* colonization, as only seven of nine birds were colonized, and the median level of colonization was reduced to 4×10^4^ CFU/gram of cecal content. The FlaA and FlpA vaccinated groups also had a reduction in the number of birds colonized, with eleven of twelve and nine of ten birds colonized, respectively. The median levels of colonization for the FlaA and FlpA vaccinated groups were 2.55×10^4^ and 4.5×10^4^ CFU/gram, respectively. Interestingly, the CadF vaccinated groups did not have any uncolonized birds, although the median level of colonization was reduced to 1.1×10^6^ CFU/gram. The CmeC vaccinated group displayed the greatest range in the level of colonization observed, and only a modest reduction in the median level of colonization. Similar results were generated in a pilot study (Figures S1 and S2).

### Antibody responses to *C. jejuni* proteins

Anti-*C. jejuni* whole cell lysate ELISAs using chicken sera revealed a significant antibody response in the *C. jejuni-*treated non-injected group compared to birds not given *C. jejuni;* (Figure 4, Panel B). Two separate 95% confidence intervals were established based on both non-injected groups. All of the vaccinated groups had significantly increased sera reactivity compared to the non-injected *C. jejuni* challenged group. The FlpA vaccinated group had the lowest levels of sera reactivity of the SECP-vaccinated groups. The level of sera reactivity did not precisely correlate to chicken colonization levels (See Table S6 for a detailed comparison).

To determine whether the antibody responses observed were specific to the antigen used for vaccination, immunoblots were performed using sera isolated from the CadF, FlaA, FlpA, and trifecta vaccinated groups (Figure S1). CmeC was not included in these analyses, as vaccination with this peptide did not reduce *C. jejuni* colonization. Chicken sera were probed against whole cell lysates of *C. jejuni* wild-type strains and the appropriate mutants. Seven of eleven birds injected with CadF had sera that reacted against protein in the whole cell lysate of *C. jejuni* wild-type strain, but not a whole cell lysate of a *C. jejuni cadF flpA* mutant. In contrast, only one FlpA vaccinated bird did not specifically react against the FlpA protein. Interestingly, we could not detect specific reactivity in the sera of FlaA vaccinated birds against FlaA in the whole cell lysates prepared from the *C. jejuni* wild-type strain. In an attempt to increase specificity, the FlaA group sera were also probed against outer-membrane extracts and purified FlaA-GST peptide. The sera of the birds were only reactive against purified FlaA-GST. The sera of all of the trifecta vaccinated birds reacted most strongly to the FlpA protein, as reactivity was not observed against proteins at the molecular weight of CadF or FlaA. Collectively, the data indicate that vaccination with FlpA resulted in the most specific antibody response.

## Discussion

A goal of *C. jejuni* researchers is to reduce the incidence of human infection and ensure a safe food supply. The level of cross-contamination between poultry products that occurs during processing, as well as between poultry and uncooked foods in the kitchen, is linked to the number of *C. jejuni* present in the ceca of the bird [10]. The likelihood of infection with *C. jejuni* correlates with the infectious dose, with as few as 500 organisms sufficient to cause disease [74]. Therefore, it should be possible to reduce the incidence of human infection by lowering the number of *C. jejuni* in birds bound for the food supply. The vaccination strategies described in this manuscript resulted in a greater than 2 log_10_ reduction in the level of *C. jejuni* colonization. According to risk analysis studies, this reduction in colonization should significantly lower the public health risk [4], [37].

In this study, we found that non-vaccinated birds colonized with high levels of *C. jejuni* produced anti-*C. jejuni* antibodies, but these antibodies did not prevent or reduce colonization. In contrast, antibodies generated prior to *C. jejuni* exposure could reduce or prevent colonization. We observed that portions of the CadF and FlpA adhesins, as well as the FlaA flagellin protein, were effective in inducing a protective immune response against *C. jejuni* colonization in chickens. FlpA and the FlpA-containing trifecta peptide provided the greatest protection against *C. jejuni* challenge, which is unsurprising given that *C. jejuni flpA* mutants fail to colonize chickens [57]. The residues required for FlpA binding to fibronectin are surface-exposed, and *C. jejuni* binding to fibronectin can be reduced by FlpA-reactive antibodies [58]. Importantly, the FlpA protein is 100% conserved amongst all *C. jejuni* isolates examined to date, indicating that this protein can be used to induce protective immunity against a wide range of *C. jejuni* isolates [57]. The sera of chickens from the trifecta group were most strongly reactive against FlpA; in fact, we could not detect specific reactivity against CadF or FlaA by immunoblot. Chickens injected with CmeC showed little protection from *C. jejuni* colonization upon challenge. It is possible that antibodies reactive against the CmeC protein did not inhibit the function of the protein. Based on the similar level of protection observed in the FlaA, FlpA, and trifecta groups, additional studies would need to be performed to determine if a combination of epitopes could enhance the efficacy of the vaccination strategy. A combination of epitopes may provide protection against a wide range of *C. jejuni* isolates that produce divergent alleles of the SECPs. Reduction of *C. jejuni* colonization of chickens was also observed in a preliminary SECP vaccination experiment (Figures S1 and S2).

We could not detect antibodies specific against FlaA in *C. jejuni* whole cell lysates. In contrast, the ELISAs revealed that FlaA vaccination did induce the production of antibodies reactive against a *C. jejuni* whole-cell lysate. More specifically, reactivity was observed against purified FlaA-GST, indicating that an immune response specific to the peptide used for vaccination was generated. A potential explanation for this apparent discrepancy is that the conformation of the FlaA protein is different in the two assays (linear versus folded). Regardless, vaccination with FlaA resulted in a decrease in the colonization of chickens by *C. jejuni*.

One limitation of this study is that the birds were challenged with a single strain of *C. jejuni,* thus it is possible that the vaccine would not be effective against *C. jejuni* strains with varying peptide sequences. The amino acid sequences of the CadF and FlpA peptides are identical amongst every strain of *C. jejuni* we have examined, while the FlaA and CmeC peptides contain variable sequences. The effectiveness of this vaccination strategy will need to be determined using a diverse panel of *C. jejuni* isolates to identify the peptides with the broadest range of protection.

An important consideration in the vaccination strategy utilized in this study is that the antigens were delivered via intramuscular injection, resulting in a robust IgY antibody response. However, as *C. jejuni* is located only in the digestive tract of chickens, only mucosal antibodies should be capable of reaching and neutralizing *C. jejuni*. While our vaccine delivery method resulted in a reduction in *C. jejuni* colonization, a delivery strategy resulting in an enhanced IgA production (*i.e.*, mucosal delivery) may further reduce the colonization of *C. jejuni.* For example, mucosal delivery of influenza vaccine results in enhanced production of mucosal antibodies compare to intramuscular injection [75]. An alternative peptide delivery method may also make the vaccination of large numbers of chickens more feasible, as the intramuscular vaccination of thousands of birds would be very labor-intensive and costly. As previously mentioned, the use of attenuated *Salmonella* to deliver antigens is currently being investigated [23], [45], [46], [48], [49]. A non-pathogen derived delivery platform is the probiotic *Lactobacillus,* which could be engineered to produce *C. jejuni* peptides. Colonization of the recombinant *Lactobacillus* in the gut could then result in delivery of the antigen to mucosal surfaces. This strategy would not require costly protein purification or injections. Regardless, the intramuscular injection of chickens with our antigens resulted in the production of a protective mucosal response as evidenced by a reduction in *C. jejuni* colonization.

The experiments described were conducted in a controlled environment using small groups of birds. It is likely that vaccination of chickens raised in other environments, such as large indoor flocks or flocks with outside access, would result in a different level of protection due to differences in bird numbers, stress levels, temperature, humidity, and the age at which the birds are first exposed to *C. jejuni.* In addition, our experimental results would not be affected by cross-contamination that would occur during industrial chicken processing. Future studies are needed to determine the efficacy of this vaccination strategy in a production environment.

We evaluated the ability of four SECPs, which included CadF, FlaA, FlpA, and CmeC, as well as a recombinant CadF-FlaA-FlpA peptide, to generate an adaptive immune response against *C. jejuni* and reduce colonization of poultry. While vaccination with CadF, FlaA, and FlpA individually reduced *C. jejuni* colonization, the trifecta peptide containing CadF-FlaA-FlpA resulted in the greatest degree of protection. Based on immunoblot analysis, the FlpA peptide resulted in the strongest and most specific antibody response, both as an individual peptide and as part of the CadF-FlaA-FlpA trifecta peptide. Additionally, a 30 amino acid portion of the FlpA peptide was found to be sufficient to generate an antibody response against neutralizing epitopes. We propose that future immune intervention strategies include FlpA, particularly since the FlpA protein is extremely well-conserved amongst *C. jejuni* strains. Although not tested in this study, the protection provided by vaccination with FlpA would likely extend to all *C. jejuni* strains. In summary, reducing the level of *C. jejuni* in the cecum by immunization of chickens with FlpA could create a safer food supply.

## Supporting Information

Figure S1 **Design for pilot chicken vaccination experiment**. The first peptide injections were given at a dose of 72 µg (GST-FlaA, GST-FlpA, GST-CjaA, GST-CmeC, and the CadF-FlaA-FlpA GST-trifecta groups) or 14.5 µg (GST-CadF peptide group only) and the booster injections were given at a dose of 240 µg (CadF-His, FlpA-His, GST-FlaA, GST-CjaA, GST-CmeC, and CadF-FlaA-FlpA GST-trifecta). Groups 1 and 2 were not injected with a *C. jejuni* CAP (NI = No injection). Each chicken within Groups 2 through 7 received a dose of 2×10^7^ CFU of *C. jejuni* at day 20. The chickens were necropsied at 27 days of age.
(TIF)

Figure S2 **Pilot chicken vaccination experiment. Panel A:** The number of CFU of *C. jejuni* was determined by serially plating diluted cecal contents on Campy-Cefex plates. The median level of colonization and interquartile range are shown for each group. The number of birds colonized in each group is shown below the graph. NI = no injection. ND = not detected; limit of detection is 10^3^ CFU/gram of cecal contents. **Panel B:** Sera was isolated from blood collected from the chickens at the time of necropsy and examined via ELISA using microtiter plates coated with a *C. jejuni* whole-cell lysate. Chicken IgY bound to the coated wells was detected with a rabbit anti-chicken IgY antibody conjugated to HRP. A 95% confidence interval was calculated based on the level of sera reactivity in the non-vaccinated, unchallenged group and non-vaccinated, non-challenged groups of chickens (dashed line).
(TIF)

Figure S3 **FlpA peptide generates a specific antibody response as judged by immunoblot.** Blood was collected at the time of necropsy and serum was used to probe whole cell lysates of a *C. jejuni* wild-type strain and mutants to determine the specificity of IgY antibodies. Numbers represent the bird within each group, and correspond to Table S6. The molecular weight of the band of interest in each panel are listed on the left in kDa. **Panel A:** Lanes A) *C. jejuni* F38011 wild-type strain; and B) *C. jejuni cadF flpA* mutant. **Panel B:** Lanes A) *C. jejuni* F38011 wild-type strain; and B) *C. jejuni cadF flpA* mutant. **Panel C:** Lanes A) *C. jejuni* F38011 wild-type strain; B) *C. jejuni flaA flaB* mutant; C) *C. jejuni* F38011 wild-type strain outer membrane fraction; and D) FlaA-GST peptide. **Panel D:** Lanes A) *C. jejuni* F38011 wild-type strain; B) *C. jejuni cadF flpA* mutant; and C) *C. jejuni flaA flaB* mutant. Only the area showing the FlpA band is shown in Panel D, as bands were not observed for FlaA or CadF.
(TIF)

Table S1 **Bacterial strains and plasmids used in this study.**
(DOC)

Table S2 **Oligonucleotides used to generate recombinant expression constructs.**
(DOC)

Table S3 **Full length (panel A), GST-tagged 90 mers (panel B), and His-tagged (panel C) proteins used in this study.**
(DOC)

Table S4 **Molecular mass of the recombinant proteins.**
(DOC)

Table S5 ***Campylobacter*** **FlaA sequences used in alignment to identify consensus sequences in surface-exposed D_2_ D_3_ domains.**
(DOC)

Table S6 **Comparison of chicken colonization, sera reactivity against** ***C. jejuni*** **whole cell lysates, and specificity of immunoblot.**
(DOC)

Materials and Methods S1 **Vaccination of chickens (pilot experiment).**
(DOC)
