# Supplementary figures and images for: Reducing Campylobacter jejuni Colonization of Poultry via Vaccination
Source: PLoS One. 2014 Dec 4;9(12):e114254. doi: 10.1371/journal.pone.0114254 (PMC4256221; doi:10.1371/journal.pone.0114254)

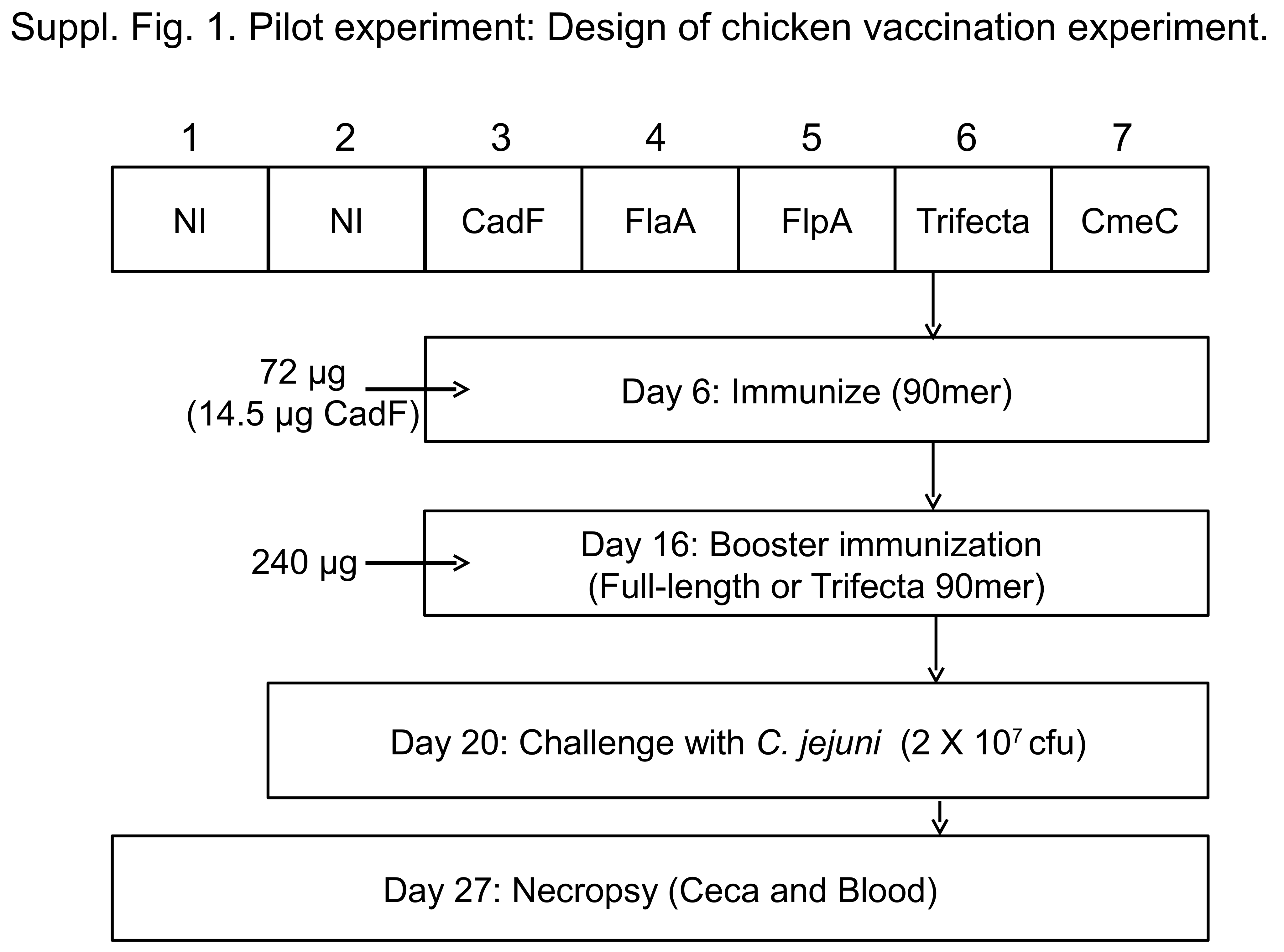

Supplement: Figure S1 — Design for pilot chicken vaccination experiment. The first peptide injections were given at a dose of 72 µg (GST-FlaA, GST-FlpA, GST-CjaA, GST-CmeC, and the CadF-FlaA-FlpA GST-trifecta groups) or 14.5 µg (GST-CadF peptide group only) and the booster injections were given at a dose of 240 µg (CadF-His, FlpA-His, GST-FlaA, GST-CjaA, GST-CmeC, and CadF-FlaA-FlpA GST-trifecta). Groups 1 and 2 were not injected with a C. jejuni CAP (NI = No injection). Each chicken within Groups 2 through 7 received a dose of 2×10^7^ CFU of C. jejuni at day 20. The chickens were necropsied at 27 days of age. (TIF) [file pone.0114254.s001.tif]

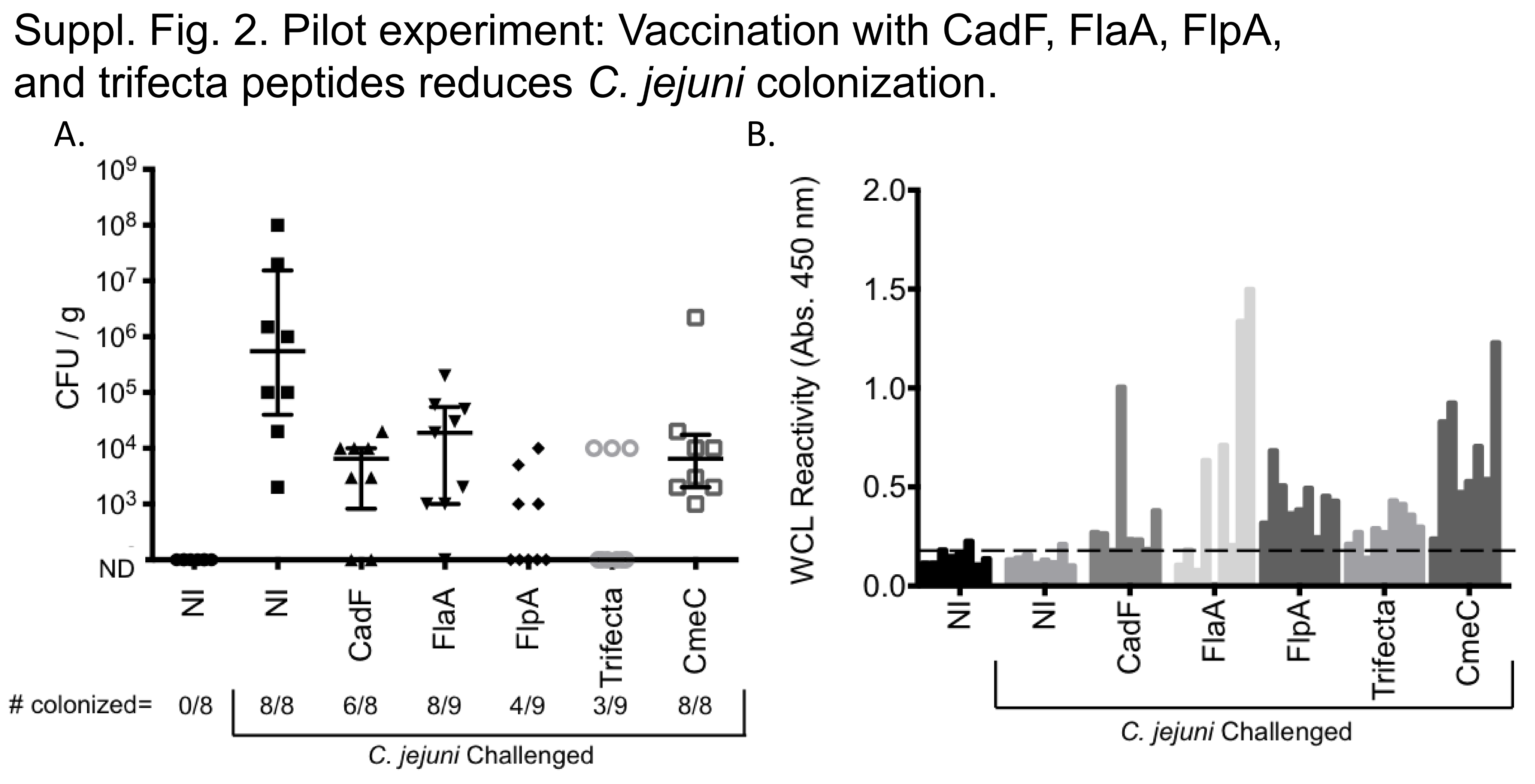

Supplement: Figure S2 — Pilot chicken vaccination experiment. Panel A: The number of CFU of C. jejuni was determined by serially plating diluted cecal contents on Campy-Cefex plates. The median level of colonization and interquartile range are shown for each group. The number of birds colonized in each group is shown below the graph. NI = no injection. ND = not detected; limit of detection is 103 CFU/gram of cecal contents. Panel B: Sera was isolated from blood collected from the chickens at the time of necropsy and examined via ELISA using microtiter plates coated with a C. jejuni whole-cell lysate. Chicken IgY bound to the coated wells was detected with a rabbit anti-chicken IgY antibody conjugated to HRP. A 95% confidence interval was calculated based on the level of sera reactivity in the non-vaccinated, unchallenged group and non-vaccinated, non-challenged groups of chickens (dashed line). (TIF) [file pone.0114254.s002.tif]

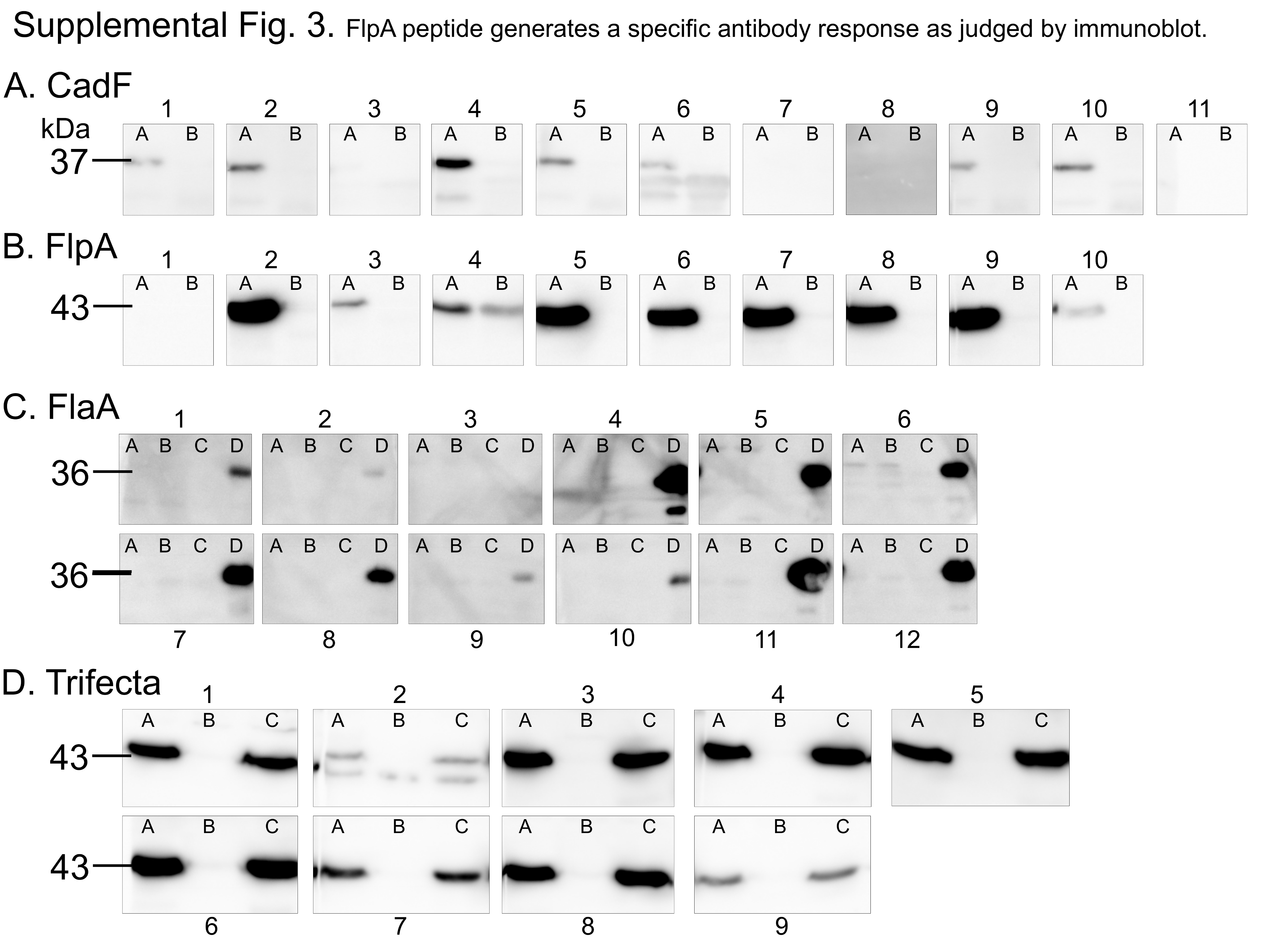

Supplement: Figure S3 — FlpA peptide generates a specific antibody response as judged by immunoblot. Blood was collected at the time of necropsy and serum was used to probe whole cell lysates of a C. jejuni wild-type strain and mutants to determine the specificity of IgY antibodies. Numbers represent the bird within each group, and correspond to Table S6. The molecular weight of the band of interest in each panel are listed on the left in kDa. Panel A: Lanes A) C. jejuni F38011 wild-type strain; and B) C. jejuni cadF flpA mutant. Panel B: Lanes A) C. jejuni F38011 wild-type strain; and B) C. jejuni cadF flpA mutant. Panel C: Lanes A) C. jejuni F38011 wild-type strain; B) C. jejuni flaA flaB mutant; C) C. jejuni F38011 wild-type strain outer membrane fraction; and D) FlaA-GST peptide. Panel D: Lanes A) C. jejuni F38011 wild-type strain; B) C. jejuni cadF flpA mutant; and C) C. jejuni flaA flaB mutant. Only the area showing the FlpA band is shown in Panel D, as bands were not observed for FlaA or CadF. (TIF) [file pone.0114254.s003.tif]
