# Supplementary material for: Reducing Campylobacter jejuni Colonization of Poultry via Vaccination
Source: PLoS One. 2014 Dec 4;9(12):e114254. doi: 10.1371/journal.pone.0114254 (PMC4256221; doi:10.1371/journal.pone.0114254)
Supplement: Table S1 — Bacterial strains and plasmids used in this study. (DOC) [file pone.0114254.s004.doc]

**Supplemental Table 1**

Bacterial strains and plasmids used in this study.

| Bacterial strains | Source |
| --- | --- |
| *C. jejuni* F38011 wild-type strain | Human clinical isolate |
| *C. jejuni* F38011 *cadF flpA* mutant | Flanagan RC, Neal-McKinney JM, Dhillon AS, Miller WG, Konkel ME (2009). Infect Immun 77: 2399-2407. |
| *C. jejuni* F38011 *flaA flaB* mutant | Neal-McKinney JM, Christensen JE, Konkel ME (2010).  Mol Microbiol 76(4):918-31 |
| *E. coli XL-1* Blue | Stratagene, Garden Grove, CA |
| *E. coli* BL21(DE3) | Novagen, Madison, WI |
| Plasmid pGEX-5X-1 | GE Healthcare, [Buckinghamshire](http://en.wikipedia.org/wiki/Buckinghamshire), UK |
| Plasmid pET-24b | Qiagen, Venlo, Netherlands |
