# Supplementary material for: Reducing Campylobacter jejuni Colonization of Poultry via Vaccination
Source: PLoS One. 2014 Dec 4;9(12):e114254. doi: 10.1371/journal.pone.0114254 (PMC4256221; doi:10.1371/journal.pone.0114254)
Supplement: Table S2 — Oligonucleotides used to generate recombinant expression constructs. (DOC) [file pone.0114254.s005.doc]

**Supplemental Table 2**

Oligonucleotides used to generate recombinant expression constructs.

| Name | Sequence 5’ to 3’ | Purpose |
| --- | --- | --- |
| CadF-90mer-EcoRI-F | ATATAGAATTCGACGATTTTTGGCTTGATCAATTAG | GST Fusion |
| CadF-90mer-XhoI-R | ATATACTCGAGAAGTCTTAAAGCCAAAGAATCACTAAG | GST Fusion |
| CadF-Full24B-EcoRI-F | ATATAGAATTCGAACAATGTAAAATTTGAAATCACTCC | 6X His-Tag |
| CadF-Full24B-XhoI-R | ATATACTCGAGTCTTAAAATAAATTTAGCATCCACTCTTC | 6X His-Tag |
| FlaA-90mer-EcoRI-F | ATATAGAATTCAAAGTTGTGATTTCAACTTCAGTTGG | GST Fusion |
| FlaA-90mer-XhoI-R | ATATACTCGAGATCGATCGAAGCTTCAACTCC | GST Fusion |
| FlaA-Full24B-EcoRI-F | ATATAGAATTCGAGACTTAGTTCAGGTCTTAGAATC | 6X His-Tag |
| FlaA-Full24B-XhoI-R | ATATACTCGAGCTGTAGTAATCTTAAAACATTTTGTTG | 6X His-Tag |
| FlpA-90mer-EcoRI-F | ATATAGAATTCGTTCAAGCTGTGACTAATTTGC | GST Fusion |
| FlpA-90mer-XhoI-R | ATATACTCGAGTAAAGCCTTGCTTGTAGAACTTAC | GST Fusion |
| FlpA-Full24B-EcoRI-F | ATATAGAATTCGTCGCTAGCTTCAAGTAAAGAGC | 6X His-Tag |
| FlpA-Full24B-XhoI-R | ATATACTCGAGCTGAGCCGCCTTAACTTTGC | 6X His-Tag |
| CmeC-90mer-EcoRI-F | ATATAGAATTCAAATCTGCAAAAGAAATTTATAGGATTAATG | GST Fusion |
| CmeC-90mer-XhoI-R | ATATACTCGAGAAGCAAGATGGTACTTGAAATTCC | GST Fusion |
| CmeC-Full24B-EcoRI-F | ATATAGAATTCGCCAAATTTAAATATTCCCGAAGCAAAC | 6X His-Tag |
| CmeC-Full24B-XhoI-R | ATATACTCGAGCGTATCTTCACTTTGCTCAAATCC | 6X His-Tag |
| Tri-Synth-EcoRI-F (Trifecta) | GACGCCGTTGAATTCCACTACGGCGCCGGCG | GST Fusion |
| Tri-Synth-XhoI-R (Trifecta) | GTTGTGCATCTCGAGTTCAATAATATAAGAATCAACACGAAAATC | GST Fusion |
