# Supplementary material for: Reducing Campylobacter jejuni Colonization of Poultry via Vaccination
Source: PLoS One. 2014 Dec 4;9(12):e114254. doi: 10.1371/journal.pone.0114254 (PMC4256221; doi:10.1371/journal.pone.0114254)
Supplement: Table S3 — Full length (panel A), GST-tagged 90 mers (panel B), and His-tagged (panel C) proteins used in this study. (DOC) [file pone.0114254.s006.doc]

**Supplemental Table 3.** Full length (panel A), GST-tagged 90mers (panel B), and His-tagged (panel C) proteins used in this study.

1. CadF (Cj1478c) from *C. jejuni* strain NCTC 11168). Highlighted in yellow is the proposed surface-exposed Fn-binding domain.

A) CadF Full-length Sequence (including signal sequence, Residues 1 - 319)

MKKIFLCLGLASVLFGADNNVKFEITPTLNYNYFEGNLDMDNRYAPGIRLGYHFDDFWLD QLEFGLEHYSDVKYTNTNKTTDITRTYLSAIKGIDVGEKFYFYGLAGGGYEDFSNAAYDN KSGGFGHYGAGVKFRLSDSLALRLETRDQINFNHANHNWVSTLGISFGFGGKKEKAVEEV ADTRATPQAKCPVEPREGALLDENGCEKTISLEGHFGFDKTTINPTFQEKIKEIAKVLDE NERYDTILEGHTDNIGSRAYNQKLSERRAKSVANELEKYGVEKSRIKTVGYGQDNPRSSN DTKEGRADNRRVDAKFILR

B) Amino acid sequence of CadF (55-144) synthesized as a GST-tagged fusion protein

GST- tag = green, vector residues= LERPHRD

MSPILGYWKIKGLVQPTRLLLEYLEEKYEEHLYERDEGDKWRNKKFELGLEFPNLPYYID

GDVKLTQSMAIIRYIADKHNMLGGCPKERAEISMLEGAVLDIRYGVSRIAYSKDFETLKV

DFLSKLPEMLKMFEDRLCHKTYLNGDHVTHPDFMLYDALDVVLYMDPMCLDAFPKLVCFK

KRIEAIPQIDKYLKSSKYIAWPLQGWQATFGGGDHPPKSDLIEGRGIPEF

DDFWLDQLEFGLEHYSDVKYTNTNKTTDITRTYLSAIKGIDVGEKFYFYGLAGGGYEDFS

NAAYDNKSGGFGHYGAGVKFRLSDSLALRL

LERPHRD

C) Amino acid sequence of CadF (19-319) synthesized as a His-tagged fusion protein

Vector residues = amino-terminal MASMTGGQQMGRDPNS and carboxy-terminal LQHHHHHH

MASMTGGQQMGRDPNS

NNVKFEITPTLNYNYFEGNLDMDNRYAPGIRLGYHFDDFWLDQLEFGLEHYSDVKYTNTN

KTTDITRTYLSAIKGIDVGEKFYFYGLAGGGYEDFSNAAYDNKSGGFGHYGAGVKFRLSD

SLALRLETRDQINFNHANHNWVSTLGISFGFGGKKEKAVEEVADTRATPQAKCPVEPREG

ALLDENGCEKTISLEGHFGFDKTTINPTFQEKIKEIAKVLDENERYDTILEGHTDNIGSR

AYNQKLSERRAKSVANELEKYGVEKSRIKTVGYGQDNPRSSNDTKEGRADNRRVDAKFIL

R

LQHHHHHH

2. FlaA (Cj1339c) sequence based on *C. jejuni* strain NCTC 11168.

A) FlaA Full-length Sequence (including signal sequence, Residues 1 - 572)

MGFRINTNVAALNAKANADLNSKSLDASLSRLSSGLRINSAADDASGMAIADSLRSQANT LGQAISNGNDALGILQTADKAMDEQLKILDTIKTKATQAAQDGQSLKTRTMLQADINRLM EELDNIANTTSFNGKQLLSGNFINQEFQIGASSNQTVKATIGATQSSKIGLTRFETGGRI STSGEVQFTLKNYNGIDDFQFQKVVISTSVGTGLGALADEINKNADKTGVRATFTVETRG IAAVRAGATSDTFAINGVKIGKVDYKDGDANGALVAAINSVKDTTGVEASIDANGQLLLT SREGRGIKIDGNIGGGAFINADMKENYGRLSLVKNDGKDILISGSNLSSAGFGATQFISQ ASVSLRESKGQIDANIADAMGFGSANKGVVLGGYSSVSAYMSSAGSGFSSGSGYSVGSGK NYSTGFANAIAISAASQLSTVYNVSAGSGFSSGSTLSQFATMKTTAFGVKDETAGVTTLK GAMAVMDIAETAITNLDQIRADIGSVQNQVTSTINNITVTQVNVKAAESQIRDVDFAAES ANYSKANILAQSGSYAMAQANSVQQNVLRLLQ

B) Amino acid sequence of FlaA (203 - 292) synthesized as a GST-tagged fusion protein

GST- tag = green, vector residues= LERPHRD

MSPILGYWKIKGLVQPTRLLLEYLEEKYEEHLYERDEGDKWRNKKFELGLEFPNLPYYID

GDVKLTQSMAIIRYIADKHNMLGGCPKERAEISMLEGAVLDIRYGVSRIAYSKDFETLKV

DFLSKLPEMLKMFEDRLCHKTYLNGDHVTHPDFMLYDALDVVLYMDPMCLDAFPKLVCFK

KRIEAIPQIDKYLKSSKYIAWPLQGWQATFGGGDHPPKSDLIEGRGIPEF

KVVISTSVGTGLGALADEINKNADKTGVRATFTVETRGIAAVRAGATSDTFAINGVKIGK

VDYKDGDANGALVAAINSVKDTTGVEASID

LERPHRD

C) Amino acid sequence of FlaA (31-572) synthesized as a His-tagged fusion protein

Vector residues = amino-terminal, MASMTGGQQMGRDPNS and carboxy-terminal, LQHHHHHH

MASMTGGQQMGRDPNS

RLSSGLRINSAADDASGMAIADSLRSQANTLGQAISNGNDALGILQTADKAMDEQLKILD

TIKTKATQAAQDGQSLKTRTMLQADINRLMEELDNIANTTSFNGKQLLSGNFINQEFQIG

ASSNQTVKATIGATQSSKIGLTRFETGGRISTSGEVQFTLKNYNGIDDFQFQKVVISTSV

GTGLGALADEINKNADKTGVRATFTVETRGIAAVRAGATSDTFAINGVKIGKVDYKDGDA

NGALVAAINSVKDTTGVEASIDANGQLLLTSREGRGIKIDGNIGGGAFINADMKENYGRL

SLVKNDGKDILISGSNLSSAGFGATQFISQASVSLRESKGQIDANIADAMGFGSANKGVV

LGGYSSVSAYMSSAGSGFSSGSGYSVGSGKNYSTGFANAIAISAASQLSTVYNVSAGSGF

SSGSTLSQFATMKTTAFGVKDETAGVTTLKGAMAVMDIAETAITNLDQIRADIGSVQNQV

TSTINNITVTQVNVKAAESQIRDVDFAAESANYSKANILAQSGSYAMAQANSVQQNVLRL

LQ

LQHHHHHH

3. FlpA (Cj1279c) sequence based on *C. jejuni* strain NCTC 11168. Highlighted in yellow is the proposed surface-exposed, Fn-binding domain.

A) FlpA Full-length Sequence (including signal sequence, Residues 1 - 411)

MMKRFRLSFYLSFLTLLLSACSVSQMNSLASSKEPAVNESLPKVESLKSLSDMSNIAFEW EPLYNENIKGFYLYRSSDENPDFKLVGTIKDKFQTHYVDTKLEPGTKYRYMMKSFNEQGQ ISEDGKVIEVSTAPRLEAVPFVQAVTNLPNRIKLIWRPHPDFRVDSYIIERTKGDDKEFK KIAEVKNRLNAEYIDSDLKPNENSSYRIIAVSFNGIKSGSSQVVSSTSKALPPQVEHLSA STDGSSKIILTWDAPTYEDFSYYKVYSTSSSFLPFSVLAKTDKNSYEDIVEGAGKSKYYK VTMVDKDGLESPMPKDGVEGKTLGNPLAPSIILAQSTSEGINLEWSDNDTRAVEYEVRRY GGEQNAVFKGIKEKRLKDVKALPGVEYSYEVIAIDSAGLRSEPSSKVKAAQ

B) Amino acid sequence of FlpA (142-231) synthesized as a GST-tagged fusion protein

GST- tag = green, vector residues= LERPHRD

MSPILGYWKIKGLVQPTRLLLEYLEEKYEEHLYERDEGDKWRNKKFELGLEFPNLPYYID

GDVKLTQSMAIIRYIADKHNMLGGCPKERAEISMLEGAVLDIRYGVSRIAYSKDFETLKV

DFLSKLPEMLKMFEDRLCHKTYLNGDHVTHPDFMLYDALDVVLYMDPMCLDAFPKLVCFK

KRIEAIPQIDKYLKSSKYIAWPLQGWQATFGGGDHPPKSDLIEGRGIPEF

VQAVTNLPNRIKLIWRPHPDFRVDSYIIERTKGDDKEFKKIAEVKNRLNAEYIDSDLKPN

ENSSYRIIAVSFNGIKSGSSQVVSSTSKAL

LERPHRD

C) Amino acid sequence of FlpA (27-411) synthesized as a His-tagged fusion protein

Vector residues = amino-terminal MASMTGGQQMGRDPNS and carboxy-terminal LQHHHHHH

MASMTGGQQMGRDPNS

NSLASSKEPAVNESLPKVESLKSLSDMSNIAFEWEPLYNENIKGFYLYRSSDENPDFKLV

GTIKDKFQTHYVDTKLEPGTKYRYMMKSFNEQGQISEDGKVIEVSTAPRLEAVPFVQAVT

NLPNRIKLIWRPHPDFRVDSYIIERTKGDDKEFKKIAEVKNRLNAEYIDSDLKPNENSSY

RIIAVSFNGIKSGSSQVVSSTSKALPPQVEHLSASTDGSSKIILTWDAPTYEDFSYYKVY

STSSSFLPFSVLAKTDKNSYEDIVEGAGKSKYYKVTMVDKDGLESPMPKDGVEGKTLGNP

LAPSIILAQSTSEGINLEWSDNDTRAVEYEVRRYGGEQNAVFKGIKEKRLKDVKALPGVE

YSYEVIAIDSAGLRSEPSSKVKAAQ

LQHHHHHH

4. CmeC (Cj0365c) sequence based on *C. jejuni* strain NCTC 11168.

A) CmeC Full-length Sequence (including signal sequence, Residues 1 - 492)

MNKIISISAIASFTLLISACSLSPNLNIPEANYSIDNKLGALSWEKENNSSITKNWWKDF
DDENLNKVVDLALKNNNDLKLAFIHMEQAAAQLGIDFSSLLPKFDGSASGSRAKTAINAP
SNRTGEVSYGNDFKMGLNLSYEIDLWGKYRDTYRASKSGFKASEYDYEAARLSVISNTVQ
TYFNLVNAYENENALKEAYKSAKEIYRINDEKFQVGAVGEYELAQARANLESMALQYNEA
KLNKENYLKALKILTSNDLNDILYKNQSYQVFNLKEFDIPTGISSTILLQRPDIGSSLEK
LTQQNYLVGVARTAFLPSLSLTGLLGFESGDLDTLVKGGSKTWNIGGNFTLPIFHWGEIY
QNVNLAKLNKDEAFVNYQNTLITAFGEIRYALVARKTIRLQYDNAQASEQSYKRIYEIAK
ERYDIGEMSLQDYLEARQNWLNAAVAFNNIKYSYANSIVDVIKAFGGGFEQSEDTSKNIK
EESKNLDMSFRE

B) Amino acid sequence of CmeC 90mer (residues 200-289 of the full length CmeC protein) synthesized as a GST-tagged fusion protein

GST- tag = green, vector residues= LERPHRD

MSPILGYWKIKGLVQPTRLLLEYLEEKYEEHLYERDEGDKWRNKKFELGLEFPNLPYYID

GDVKLTQSMAIIRYIADKHNMLGGCPKERAEISMLEGAVLDIRYGVSRIAYSKDFETLKV

DFLSKLPEMLKMFEDRLCHKTYLNGDHVTHPDFMLYDALDVVLYMDPMCLDAFPKLVCFK

KRIEAIPQIDKYLKSSKYIAWPLQGWQATFGGGDHPPKSDLIEGRGIPEF

KSAKEIYRINDEKFQVGAVGEYELAQARANLESMALQYNEAKLNKENYLKALKILTSNDL

NDILYKNQSYQVFNLKEFDIPTGISSTILL

LERPHRD

C) Amino acid sequence of CmeC (24-492) synthesized as a His-tagged fusion protein

Vector residues = amino-terminal MASMTGGQQMGRDPNS and carboxy-terminal LQHHHHHH

MASMTGGQQMGRDPNS

PNLNIPEANYSIDNKLGALSWEKENNSSITKNWWKDFDDENLNKVVDLALKNNNDLKLAF

IHMEQAAAQLGIDFSSLLPKFDGSASGSRAKTAINAPSNRTGEVSYGNDFKMGLNLSYEI

DLWGKYRDTYRASKSGFKASEYDYEAARLSVISNTVQTYFNLVNAYENENALKEAYKSAK

EIYRINDEKFQVGAVGEYELAQARANLESMALQYNEAKLNKENYLKALKILTSNDLNDIL

YKNQSYQVFNLKEFDIPTGISSTILLQRPDIGSSLEKLTQQNYLVGVARTAFLPSLSLTG

LLGFESGDLDTLVKGGSKTWNIGGNFTLPIFHWGEIYQNVNLAKLNKDEAFVNYQNTLIT

AFGEIRYALVARKTIRLQYDNAQASEQSYKRIYEIAKERYDIGEMSLQDYLEARQNWLNA

AVAFNNIKYSYANSIVDVIKAFGGGFEQSEDTSKNIKEESKNLDMSFRE

LQHHHHHH

5. CadF-FlaA-FlpA trifecta, 90mer [each fragment is a 30 residues, CadF sequence (residues 127-156) based on *C. jejuni* strain NCTC 11168, FlaA sequence is a consensus sequence based on 8 *C. jejuni* strains (See Supplemental Figure 1, residues 278-307 based on the FlaA alignment), and FlpA sequence (141-170) based on *C. jejuni* strain NCTC 1168].

GST- tag = green, vector residues= LERPHRD

MSPILGYWKIKGLVQPTRLLLEYLEEKYEEHLYERDEGDKWRNKKFELGLEFPNLPYYID

GDVKLTQSMAIIRYIADKHNMLGGCPKERAEISMLEGAVLDIRYGVSRIAYSKDFETLKV

DFLSKLPEMLKMFEDRLCHKTYLNGDHVTHPDFMLYDALDVVLYMDPMCLDAFPKLVCFK

KRIEAIPQIDKYLKSSKYIAWPLQGWQATFGGGDHPPKSDLIEGRGIPEF

HYGAGVKFRLSDSLALRLETRDQINFNHANGGSGINAVKDTTGVEASIDANGQLVLTS

ADGRGIGGSGFVQAVTNLPNRIKLIWRPHPDFRVDSYIIE

LERPHRD

CadF 30mer 127-156

HYGAGVKFRLSDSLALRLETRDQINFNHAN

FlaA synthetic 278-307 (consensus sequence)

INAVKDTTGVEASIDANGQLVLTSADGRGI

FlpA 141-170

FVQAVTNLPNRIKLIWRPHPDFRVDSYIIE

Linker = spacer

GGSG
