# Supplementary material for: Reducing Campylobacter jejuni Colonization of Poultry via Vaccination
Source: PLoS One. 2014 Dec 4;9(12):e114254. doi: 10.1371/journal.pone.0114254 (PMC4256221; doi:10.1371/journal.pone.0114254)
Supplement: Table S4 — Molecular mass of the recombinant proteins. (DOC) [file pone.0114254.s007.doc]

**Supplemental Table 4**. Molecular mass of the recombinant proteins**.**

**A.** Calculated molecular mass of the 90mer protein

90 mer Apparent *M*_r_ + N-terminal GST tag Cal. *M*_r_ + N-terminal GST tag

CadF 36 kDa 37,783.20

FlaA 36 kDa 36,710.19

FlpA 36 kDa 37,907.69

CmeC 36 kDa 38,005.84

CadF-FlaA-FlpA trifecta 36 kDa 38,247.91

**B.** Calculated molecular mass of the full length protein minus the signal peptide/sequence

Full length Apparent *M*_r_ + C-terminal His tag Cal. *M*_r_ + C-terminal His tag

CadF 32 kDa 36,802.63

FlaA 60 kDa 58,663.16

FlpA 42 kDa 45,782.34

CmeC 50 kDa 55,766.31
