# Supplementary material for: Reducing Campylobacter jejuni Colonization of Poultry via Vaccination
Source: PLoS One. 2014 Dec 4;9(12):e114254. doi: 10.1371/journal.pone.0114254 (PMC4256221; doi:10.1371/journal.pone.0114254)
Supplement: Table S5 — Campylobacter FlaA sequences used in alignment to identify consensus sequences in surface-exposed D2 D3 domains. (DOC) [file pone.0114254.s008.doc]

**Supplemental Table 5**. *Campylobacter* FlaA sequences used in alignment to identify consensus sequences in surface-exposed D_2_ D_3_ domains.

>gi|120253|sp|P27053|FLAA_CAMCO Flagellin A [Campylobacter coli]*

MGFRINTNVAALNAKANSDLNSRALDQSLSRLSSGLRINSAADDASGMAIADSLRSQANTLGQAISNGNDALGILQTADKAMDEQLKILDTIKTKATQAAQDGQSLKTRTMLQADINRLMEELDNIANTTSFNGKQLLSGGFTNQEFQIGSSSNQTIKASIGATQSSKIGVTRFETGSQSFSSGTVGLTIKNYNGIEDFKFDSVVISTSVGTGLGALAEEINRNADKTGIRATFDVKSVGAYAIKAGNTSQDFAINGVVIGKVDYSDGDENGSLISAINAVKDTTGVQASKDENGKLVLTSADGRGIKITGSIGVGAGILHTENYGRLSLVKNDGRDINISGTGLSAIGMGATDMISQSSVSLRESKGQISAANADAMGFNAYNGGGAKQIIFASSIAGFMSQAGSGFSAGSGFSVGSGKNYSAILSASIQIVSSARSISSTYVVSTGSGFSAGSGNSQFAALRISTVSAHDETAGVTTLKGAMAVMDIAETAITNLDQIRADIGSVQNQITSTINNITVTQVNVKSAESQIRDVDFASESANYSKANILAQSGSYAMAQANSSQQNVLRLLQ

>gi|120254|sp|P22251|FLA2_CAMJE Flagellin A*

MGFRINTNVAALNAKANSDLNAKSLDASLSRLSSGLRINSAADDASGMAIADSLRSQANTLGQAISNGNDALGILQTADKAMDEQLKILDTIKTKATQAAQDGQSLKTRTMLQADINKLMEELDNIANTTSFNGKQLLSGNFTNQEFQIGASSNQTVKATIGATQSSKIGVTRFETGAQSFTSGVVGLTIKNYNGIEDFKFDNVVISTSVGTGLGALAEEINKSADKTGVRATYDVKTTGVYAIKEGTTSQDFAINGVTIGKIEYKDGDGNGSLISAINAVKDTTGVQASKDENGKLVLTSADGRGIKITGDIGVGSGILANQKENYGRLSLVKNDGRDINISGTNLSAIGMGTTDMISQSSVSLRESKGQISATNADAMGFNSYKGGGKFVFTQNVSSISAFMSAQGSGFSRGSGFSVGSGKNLSVGLSQGIQIISSAASMSNTYVVSAGSGFSSGSGNSQFAALKTTAANTTDETAGVTTLKGAMAVMDIAETAITNLDQIRADIGSIQNQVTSTINNITVTQVNVKAAESQIRDVDFASESANYSKANILAQSGSYAMAQANSSQQNVLRLLQ

>gi|13111556|gb|AAK12373.1| flagellin A [Campylobacter jejuni]*

MGFRINTNGAALNAKANSDLNAKSLDSSLARLSSGLRINSAADDASGMAIADSLRSQANTLGQAISNGNDALGILQTADKAMDEQLKILDTIKTKATQAAQDGQSLKTRTMLQADINKLMEELDNIANTTSFNGKQLLSGGFTNQEFQIGSSSNQTVKATIGATQSSKIGVTRFETGSQSFTSGVVGLTIKNYNGIEDFKFDNVVISTSVGTGLGALAEEINKNADKTGVRATYDVKTTGAYAIKAGTTSQDFAINGVIIGKVDYKDGDNNGSLISAINAVKDTTGVQASKDENGKLVLTSADGRGIKITGDIGVGSGILSTQKENYGRLSLVKNDGRDINVSGTGLSAIGMGAADMISQASVSLRESKGQISAANADAMGFNSYNGGGAKQILQASSISAFMSQAGSGFSAGSGFSAGSGKGYSTILSGSVQIVSSTASMSSTYVISEGSGFSAGSGNSQFAALKTSTVSAHEATAGVTTLKGAMAVMDIAETAITNLDQIRADIGSIQNQVTSTINNITVTQVNVKSAESQIRDVDFASESANYSKANILAQSGSYAMAQANSSQQNVLRLLQ

>gi|13111566|gb|AAK12378.1| flagellin A [Campylobacter jejuni]*

MGFRINTNGAALNAKANSDLNAKSLDSSLARLSSGLRINSAADDASGMAIADSLRSQANTLGQAISNGNDALGILQTADKAMDEQLKILDTIKTKATQAAQDGQSLKTRTMLQADINKLMEELDNIANTTSFNGKQLLSGGFTNQEFQIGSSSNQTVKATIGATQSSKIGVTRFETGSQSFTSGVVGLTIKNYNGIEDFKFDNVVISTSVGTGLGALAEEINKNADKTGVRATYDVKTTGAYAIKAGTTSQDFAINGVIIGKVDYKDGDNNGSLISAINAVKDTTGVQASKDENGKLVLTSADGRGIKITGDIGVGSGILSAQKENYGRLSLVKNDGRDINVSGTGLSAIGMGAADMISQASVSLRESKGQISAANADAMGFNSYNGGGAKQILQVQASSISAFMSQAGSGFSAGSGFSAGSGKGYSTILSGSVQIVSSTASMSSTYVISAGSGFSVGSGNSQFAALKTSTVSAHEATAGVTTLKGAMAVMDIAETAITNLDQIRADIGSIQNQVTSTINNITVTQVNVKSAESQIRDVDFASESANYSKANILAQSGSYAMAQANSSQQNVLRLLQ

>gi|15792662|ref|NP_282485.1| flagellin [Campylobacter jejuni subsp. jejuni NCTC 11168]*

MGFRINTNVAALNAKANADLNSKSLDASLSRLSSGLRINSAADDASGMAIADSLRSQANTLGQAISNGNDALGILQTADKAMDEQLKILDTIKTKATQAAQDGQSLKTRTMLQADINRLMEELDNIANTTSFNGKQLLSGNFINQEFQIGASSNQTVKATIGATQSSKIGLTRFETGGRISTSGEVQFTLKNYNGIDDFQFQKVVISTSVGTGLGALADEINKNADKTGVRATFTVETRGIAAVRAGATSDTFAINGVKIGKVDYKDGDANGALVAAINSVKDTTGVEASIDANGQLLLTSREGRGIKIDGNIGGGAFINADMKENYGRLSLVKNDGKDILISGSNLSSAGFGATQFISQASVSLRESKGQIDANIADAMGFGSANKGVVLGGYSSVSAYMSSAGSGFSSGSGYSVGSGKNYSTGFANAIAISAASQLSTVYNVSAGSGFSSGSTLSQFATMKTTAFGVKDETAGVTTLKGAMAVMDIAETAITNLDQIRADIGSVQNQVTSTINNITVTQVNVKAAESQIRDVDFAAESANYSKANILAQSGSYAMAQANSVQQNVLRLLQ

>gi|3290024|gb|AAC25637.1| flagellin A [Campylobacter jejuni]*

MGFRINTNVAALNAKANSDLNAKSLDASLSRLSSGLRINSAADDASGMAIADSLRSQANTLGQAISNGNDALGILQTADKAMDEQLKILDTIKTKATQAAQDGQSLKTRTMLQADINKLMEELDNIANTTSFNGKQLLSGNFTNQEFQIGASSNQTVKATIGATQSSKIGVTRFETGAQSFTSGVVGLTIKNYNGIEDFKFDNVVISTSVGTGLGALAEEINKSADKTGVRATYDVKTTGVYAIKEGTTSQDFAINGVTIGKIEYKDGDGNGSLISAINAVKDTTGVQASKDENGKLVLTSADGRGIKITGDIGVGSGILANQKENYGRLSLVKNDGRDINISGTNLSAIGMGTTDMISQSSVSLRESKGQISATNADAMGFNSYKGGGKFVFTQNVSSISAFMSAQDSGFSRGSGFSVGSGKNLSVGLSQGIQIISSAASMSNTYVVSAGSGFSSGSGNSQFAALKTTAANTTDETAGVTTLKGAMAVMDIAETAITNLDQIRADIGSIQNQVTSTINNITVTQVNVKAAESQIRDVDFASESANYSKANILAQSGSYAMAQANSSQQNVLRLLQ

>gi|3290026|gb|AAC25638.1| flagellin A [Campylobacter jejuni]*

MGFRINTNVAALNAKANADLNSKSLDASLSRLSSGLRINSAADDASGMAIADSLRSQANTLGQAISNGNDALGILQTADKAMDEQLKILDTIKTKATQAAQDGQSLKTRTMLQANINRLMEELDNIANTTSFSGKQLLSGNFINQEFQIGASSNQTVKATIGATQSSKIGLTRFETGERISSSGEVQFTLKNYNGIDDFKFQKVVISTSVGTGLGALADEINKNADKTGVRATFTVETRGMAAVRAGTTSDDFAINGVKIGKVDYKDGDSNGALVSAINSVKDTTGVEASIDANGQLLLTSREGRGIKIEGNIGGGAFINASMKENYGRLSLVKNDGKDILISGSNLSSAGFGATQFTSQASVSLRESKGQIDANIADAMGFGSVNKGVKLSGFSTVTAYMSSAGSGFSAGSGYSVGSGKNYSTSISGIAVAFSSGSGLSAEYNVSAGSGFSSQSGLSQFATMKTSVGNSLGVKDETAGVTTLKGAMAVMDIAETAITNLDQIRADIGSVQNQVTSTINNITVTQVNVKAAESQIRDVDFAAESANYSKANILAQSGSYAMAQANSVQQNVLRLLQ

>gi|3290028|gb|AAC25639.1| flagellin A [Campylobacter jejuni]*

MGFRINTNGAALNAKANADLNSKSLDASLSRLSSGLRINSAADDASGMAIADSLRSQANTLGQAISNGNDALGILQTADKAMDEQLKILDTIKTKATQAAQDGQSLKTRTMLQADINRLMEELDNIANTTSFNGKQLLSGNFINQEFQIGASSNQTVKATIGATQSSKIGLTRFETGGRISSSGEVQFTLKNYNGIDDFQFQKVVISTSVGTGLGALADEINKNADKTGVRATFTVETRGMAAVRAGTTSDDFAINGVKIGKVAYEDGDANGALVSAINSVKDTTGVEASIDANGQLLLTSREGRGIKIEGSIGGGAFINKDMMENYGRLSLVKNDGKDISISGTGLSFTGFGASNFISQVSVSLRESKGQLDANTADAMGFGSVNKGLVLAASSIADYMSAEGSGFSAGSGYSVGSGKGYSATLTANAIAISSASAISKIYNVSQGSGFSSGSTLSQFATMKTSAGNSLGAKDETAGVTTLKGAMAVMDIAETAITNLDQIRADIGSVQNQVTSTINNITVTQVNVKAAESQIRDVDFAAESANYSKANILAQSGSYAMAQANSVQQNVLRLLQ

>gi|3290032|gb|AAC25641.1| flagellin A [Campylobacter jejuni]*

MGFRINTNVAALNAKANADLNSKSLDASLSRLSSGLRINSAADDASGMAIADSLRSQANTLGQAISNGNDALGILQTADKAMDEQLKILDTIKTKATQAAQDGQSLKTRTMLQADINRLMEELDNIANTTSFNGKQLLSGNFINQEFQIGASSNQTVKATIGATQSSKIGLTRFETGGRISSSGEVQFTLKNYNGIDDFQFQKVVISTSVGTGLGALADEINKNADKTGVRATFTVETRGIAAVRAGATSDTFAINGVKIGKVDYKDGDANGALVAAINSVKDTTGVEASIDANGQLLLTSREGRGIKIDGNIGGGAFINADMKENYGRLSLVKNDGKDILISGSNLSSAGFGATQFISQASVSLRESKGQIDANIADAMGFGSANKGVVLGGYSSVSAYMSSAGSGFSSGSGYSVGSGKNYSTGFANAIAISAASQLSTVYNVSAGSGFSSGSTLSQFATKKTTAFGVKDETAGVTTLKGAMAVMDIAETAITNLDQIRADIGSVQNQVTSTINNITVTQVNVKAAESQIRDVDFAAESANYSKANILAQSGSYAMAQANSVQQNVLRLLQ

>gi|3290036|gb|AAC25643.1| flagellin A [Campylobacter jejuni]*

MGFRINTNVSALNAKPNSDLNAKSLDASLSRLSSGLRINSAADDASGMAIADSLRSQTNTLGQAISNGNDALGILQTADKAMDEQLKILDTIKTKATQAAQDGQSLKTRTMLQADINRLMEELDNIANTTSFNGKQLLSGNFTNQEFQIGASSNQTIKATIGATQSSKIGVTRFETGAQSFTSGVVGLTIKNYNGIEDFKFDNVVISTSVGTGLGALAEEINKSADKTGVRATYDVKTTGVYAIKEGTTSQNFAINGVVIGQINYKDGDNNGQLVSAINAVKDTTGVQASKDENGKLVLTSADGRGIKITGDIGVGSGILANQKENYGRLSLVKNDGRDINISGTNLSAIGMGTTDMISQSSVSLRESKGQISATNADAMGFNSYKGGGKFVFTQNVSSISAFMSAQGSGFSRGSGFSVGSGKNLSVGLSQGIQIISSAASMSNTYVVSAGSGFSSGSGNSQFGVLKTTAANTTDETAGVTTLKGAMAGMDIAETAITNLDQIRADIGSIQNQVTSTINNITVTQVNVKAAESQIRDVDFSSESANYSKANILAQSGSYSMAQANSSQQNVLRLLQ

>gi|3290038|gb|AAC25644.1| flagellin A [Campylobacter jejuni]*

MGFRINTNGAALNAKANSDLNAKSLDASLSRLSSGLRINSAADDASGMAIADSLRSQANTLGQAISNGNDALGILQTADKAMDEQLKILDTIKTKATQAAQDGQSLKTRTMLQADINKLMEELDNIANTTSFNGKQLLSGNFTNQEFQIGASSNQTVKATIGATQSSKIGVTRFETGAQSFTSGVVGLTIKNYNGIEDFKFDNVVISTSVGTGLGALAEEINKSADKTGVRATYDVKTTGVYAIKEGTTSQEFAINGVTIGKIEYKDGDGNGSLISAINAVKDTTGVQASKDENGKLVLTSADGRGIKITGDIGVGSGILANQKENYGRLSLVKNDGRDINISGTNLSAIGMGTTDMISQSSVSLRESKGQISATNADAMGFNSYKGGGKFVFTQNVSSISAFMSAQGSGFSRGSGFSVGSGKNLSVGLSQGIQIISSAGSMSNTYVVSAGSGFSSGSGNSQFAALKTTAANTTDETAGVTTLKGAMAVMDIAETAITNLDQIRADIGSIQNQVTSTINNITVTQVNVKAAESQIRDVDFASESANYSKANILAQSGSYAMAQANSSQQNVLRLLQ

>gi|3290040|gb|AAC25645.1| flagellin A [Campylobacter jejuni]*

MGFRINTNVAALNAKANADLNSKSLDASLSRLSSGLRINSAADDASGMAIADSLRSQANTLGQAISNGNDALGILQTADKAMDEQLKILDTIKTKATQAAQDGQSLKTRTMLQADINRLMEELDNIANTTSFNGKQLLSGNFINQEFQIGASSNQTVKATIGATQSSKIGLTRFETGGRISSSGEVQFTLKNYNGIDDFQFQKVVISTSVGTGLGALADEINKNADKTGVRATFTVETRGIAAVRAGATSDDFAINGVKIGKVDYKDGDANGALVAAINSVKDTTGVEASIDANGQLLLTSREGRGIKIDGNIGGGAFINADMKENYGRLSLVKNDGKDILISGSNLSSAGFGATQFISQASVSLRESKGQIDANIADAMGFGSANKGVVLGGYSSVSAYMSSAGSGFSSGSGYSVGSGKNYSTGFANAIAISAASQLSTVYNVSAGSGFSSGSTLSQFATMKTTAFGVKDETAGVTTLKGAMAVMDIAETAITNLDQIRADIGSVQNQVTSTINNITVTQVNVKAAESQIRDVDFAAESANYSKANILAQSGSYAMAQANSVQQNVLRLLQ

>gi|3290044|gb|AAC25647.1| flagellin A [Campylobacter jejuni]*

MGFRINTNVAALNAKANSDLNSKALDQSLARLSSGLRINSAADDASGMAIADSLRSQASTLGQAISNGNDALGILQTADKAMDEQLKILDTIKTKATQAAQDGQSLKTRTMLQADINRLMEELDNIANTTSFNGKQLLSGNFINQEFQIGASSNQTVKTTIGATQSSKIGLTRFETGGRISESGEVQFTLKNYNGIDDFKFQKVVISTSVGTGLGALADEINKNADKTGVRATFTVETRGMGAVREGTTSDDFTINGVKIGKVEYKDGDSNGALVAAINSVKDTTGVEASIDVNGQLLLTSREGRGIKIEGDIGRGAFINPNMKENYGRLSLVKNDGKDILISGTGLTATGFGVNSFISQASVSLRESKGQIDANVADAMGFNSVDKGNILGGFSSVSSYMSSAGSGFSSGSGFSVGSGKNYSTGFANVVVVSAISQMSAVYNISAGSGFSSQSGLSQFATMKTSVGNTLGVKDETAGVTTLKGAMAVMDIAETAITNLDQIRADIGSVQNQITSTINNITVTQVNVKSAESQIRDVDFAAESANYSKANILAQSGSYAMAQANSSQQNVLRLLQ

>gi|3290046|gb|AAC25648.1| flagellin A [Campylobacter jejuni]*

MGFRINTNVAALNAKANSDLNAKSLDASLSRLSSGLRINSAADDASGMAIADSLRSQANTLGQAISNGNDALGILQTADKAMDEQLKILDTIKTKATQAAQDGQSLKTRTMLQADINKLMEELDNIANTTSFNGKQLLSGNFTNQEFQIGASSNQTVKATIGATQSSKIGVTRFETGAQSFTSGVVGLTIKNYNGIEDFKFDNVVISTSVGTGLGALAEEINKSADKTGVRATYDVKTTGVYAIKEGTTSQNFAINGVTIGKIEYKDGDGNGSLISAINAVKDTTGVQASKDENGKLVLTSADGRGIKITGDIGVGSGILANQKENYGRLSLVKNDGRDINISGTNLSAIGMGTTDMISQSSVSLRESKGQISATNADAMGFNSYKGGGKFVFTQNVSSISAFMSAQGSGFSRGSGFSVGSGKNLSVGLSQGIQIISSAASMSNTYVVSAGSGFSSGSGNSQFAALKTTAANTTDETAGVTTLKGAMAVMDIAETAITNLDQIRADIGSIQNQVTSTINNITVTQVNVKAAESQIRDVDFASESANYSKANILAQSGSHAMAQANSSQQNVLRLLQ

>gi|3290048|gb|AAC25649.1| flagellin A [Campylobacter jejuni]*

MGFRINTNVAALNAKANADLNSKSLDASLSRLSSGLRINSAADDASGMAIADSLRSQANTLGQAISNGNDALGILQTADKAMDEQLKILDTIKTKATQAAQDGQSLKTRTMLQADINRLMEELDNIANTTSFNGKQLLSGNFINQEFQIGASSNQTVKATIGATQSSKIGLTRFETGGRISSSGEVQFTLKNYNGIDDFQFQKVVISTSVGTGLGALADEINKNADKTGVRATFTVETRGIAAVRAGATSDDFAINGVKIGKVDYKDGDANGALVAAINSVKDTTGVEASIDANGQLLLTSREGRGIKIDGNIGGGAFINADMKENYGRLSLVKNDGKDILISGSNLSSAGFGATQFISQASVSLRESKGRFDANIADAMGFGSANKGVVLGGYSSVSAYMSSAGSGFSSGSGYSVGSGKNYSTGFAKAIAISAASQLSTVYNVSAGSGFSSGSTLSQFATMKTTAFGVKDETAGVTTLKGAMAVMDIAETATTNLDQIRADIGSVQNQVTSTINNITVTQVNVKAAESQIRDVDFAAESANYSKANILAQSGSYAMAQANSVQQNVLRLLQ

>gi|3290050|gb|AAC25650.1| flagellin A [Campylobacter jejuni]*

MGFRINTNVAALNAKANADLNSKSLDASLSRLSSGLRINSAADDASGMAIADTLRSQANTLGQAISNGNDAIGILQTADKAMDEQLKILDTIKTKATQAAQDGQSLKTRTMLQADINRLMEELDNIANTTSFNGKQLLSGNFINQEFQIGASSNQTVKATIGATQSSKIGLTRFETGGRISTSGEVQFTLKNYNGIDDFQFQKVVISTSVGTGLGALADEINKNADKTGVRATFTVETRGIAAVRAGATSDTFAINGVKIGKVDYKDGDANGALVAAINSVKDTTGVEASIDANGQLLLTSREGRGIKIDGNIGGGAFINADMKENYGRLSLVKNDGKDILISGSNLSSAGFGATQFISQASVSLRESKGQIDANIADAMGFGSANKGVVLGGYSSVSAYMSSAGSGFSSGSGYSVGSGKNYSTGFANAIAISAASQLSTVYNVSAGSGFSSGSTLSQFATMKTTAFGVKDETAGVTTLKGAMAVMDIAETATTNLDQIRADIGSVQNQVTSTINNITVTQVNVKAAESQIRDVDFAAESANYSKANILAQSGSYAMAQANSVHQNVLRLLQ

>gi|540931|pir||S41310 flagellin A - Campylobacter jejuni *

MGFRINTNVAALNAKANADLNSKSLDASLSRLSSGLRINSAADDASGMAIADSLRSQANTLGQAISNGNDALGILQTADKAMDEQLKILDTIKTKATQAAQDGQSLKTRTMLQADINRLMEELDNIANTTSFNGKQLLSGNFINQEFQIGASSNQTVKASIGATQSSKIGLTRFETGSRISVGGEVQFTLKNYNGIDDFKFQKVVISTSVGTGLGALRDEINKNADKTGVRATFTVETRGMGAVRAGATSDDFAINGVKIGKVDYKDGDANGALVSAINSVKDTTGVEASIDENGKLLLTSREGRGIKIEGNIGRGAFINPNMLENYGRLSLVKNDGKDILISGTNLSAIGFGTGNMISQASVSLRESKGQIDANVADAMGFNSANKGNILGGYSSVSAYMSSTGSGFSSGSGFSVGSGKNYSTGFANTIAISAASQLSAVYNVSAGSGFSSGSNLSQFATMKTSAGNTLGVKDETAGVTTLKGAMAVMDIAETAITNLDQIRADIGSVQNQVTSTINNITVTQVNVKAAESQIRDVDFAAESANYSKANILAQSGSYAMAQANSVQQNVLRLLQ

>gi|57168249|ref|ZP_00367388.1| flagellin (flaA) [Campylobacter coli RM2228]*

MGFRINTNVAALNAKANADLNSKSLDASLSRLSSGLRINSAADDASGMAIADSLRSQANTLGQAISNGNDALGILQTADKAMDEQLKILDTIKTKATQAAQDGQSLKTRTMLQADINRLMEELDNIANTTSFNGKQLLSGNFINQEFQIGASSNQTVKATIGATQSSKIGLTRFETGGRISTSGEVQFTLKNYNGIDDFQFQKVVISTSVGTGLGALADEINKNADKTGVRATFTVETRGMAAVRAGTTSDDFAINGVKIGKVEYKDGDSNGALVAAINSVKDTTGVEASIDANGQLLLSSREGRGIKIEGSIGGGAFINKDMMENYGRLSLVKNDGKDILVSGTGLESAGFGAGNFISQASVSLRESKGQLNANIADAMGFGSVNKGVILAGASSVSAYMSAAGSGFSAGSGYSAGSGKNYSAVISANAVVISNTSAISKIYDVSAGSGFSSGSTLSQFATMKTSAGNSLGAKDETAGVTTLKGAMAVMDIAETAITNLDQIRADIGSVQNQVTSTINNITVTQVNVKAAESQIRDVDFAAESANYSKANILAQSGSYAMAQANSVQQNVLRLLQ

>gi|6224680|gb|AAF05902.1|AF140252_1 flagellin A; FlaA [Campylobacter jejuni]*

MGFRINTNVAALNAKANSDLNSRALDQSLSRLSSGLRINSAADDASGMAIADSLRSQANTLGQAISNGNDALGILQTADKAMDEQLKILDTIKTKATQAAQDGQSLKTRTMLQADINRLMEELDNIANTTSFNGKQLLSGGFTNQEFQIGSSSNQTIKASIGATQSSKIGVTRFETGSQSFSSGTVGLTIKNYNGIEDFKFDSVVISTSVGTGLGALAEEINRNADKTGIRATFDVKSVGAYAIKAGNTSQDFAINGVVIGQINYNDGDNNGQLISAINAVKDTTGVQASKDENGKLVLTSADGRGIKITGSIGVGAGILHTENYGRLSLVKNDGRDINISGTGLSAIGMGATDMISQSSVSLRESKGQISAANADAMGFNSYKGGGKFVFTQNVSSISAFMSAQGSGFSRGSGFSVGSGKNLSVGLSQGIQIISSAASMSNTYVVSAGSGFSSGSGNSQFAALKTTAANTTDETAGVTTLKGAMAVMDIAETAITNLDQIRADIGSIQNQVTSTINNITVTQVNVKAAESQIRDVDFASESANYSKANILAQSGSYAMAQANSSQQNVLRLLQ

>gi|86150126|ref|ZP_01068354.1| flagellin subunit protein FlaA [Campylobacter jejuni subsp. jejuni CF93-6]*

MGFRINTNVAALNAKANADLNSKSLDASLSRLSSGLRINSAADDASGMAIADSLRSQANTLGQAISNGNDALGILQTADKAMDEQLKILDTIKTKATQAAQDGQSLKTRTMLQADINRLMEELDNIANTTSFNGKQLLSGNFINQEFQIGASSNQTVKATIGATQSSKIGLTRFETGGRISTSGEVQFTLKNYNGIDDFQFQKVVISTSVGTGLGALADEINKNADKTGVRATFTVETRGIAAVRAGATSDTFAINGVKIGKVDYKDGDANGALVAAINSVKDTTGVEASIDANGQLLLTSREGRGIKIDGNIGGGAFINADMKENYGRLSLVKNDGKDILISGSNLSSAGFGATQFISQASVSLRESKGQIDANIADAMGFGSANKGVVLGGYSSVSAYMSSAESGFSSGSGYSVGSGKNYSTGFANAIAISAASQLSTVYNVSAGSGFSSGSTLSQFATMKTTAFGVKDETAGVTTLKGAMAVMDIAETAITNLDQIRADIGSVQNQVTSTINNITVTQVNVKAAESQIRDVDFAAESANYSKANILAQSGSYAMAQANSVQQNVLRLLQ

>gi|86151038|ref|ZP_01069254.1| flagellin subunit protein FlaA [Campylobacter jejuni subsp. jejuni 260.94]*

MGFRINTNVAALNAKANSDLNAKSLDSSLARLSSGLRINSAADDASGMAIADSLRSQANTLGQAISNGNDALGILQTADKAMDEQLKILDTIKTKATQAAQDGQSLKTRTMLQADINKLMEELDNIANTTSFNGKQLLSGGFTNQEFQIGSSSNQTVKATIGATQSSKIGVTRFETGSQSFTSGVVGLTIKNYNGIEDFKFDNVVISTSVGTGLGALAEEINKNADKTGVRATYDVKTTGAYAIKAGTTSQDFAINGVIIGKVDYKDGDNNGSLISAINAVKDTTGVQASKDENGKLVLTSADGRGIKITGDIGVGSGILSAQKENYGRLSLVKNDGRDINVSGTGLSAIGMGAADMISQASVSLRESKGQISAANADAMGFNSYNGGGAKQILQVQASSISAFMSQAGSGFSAGSGFSAGSGKGYSTILSGSVQIVSSTASMSSTYVISAGSGFSVGSGNSQFAALKTSTVSAHEATAGVTTLKGAMAVMDIAETAITNLDQIRADIGSIQNQVTSTINNITVTQVNVKSAESQIRDVDFASESANYSKANILAQSGSYAMAQANSSQQNVLRLLQ

>gi|86152946|ref|ZP_01071151.1| flagellin A [Campylobacter jejuni subsp. jejuni HB93-13]*

MGFRINTNIGALNAHANSVVNANELDKSLSRLSSGLRINSAADDASGMAIADSLRSQAATLGQAINNGNDAIGILQTADKAMDEQLKILDTIKTKATQAAQDGQSLKTRTMLQADINRLMEELDNIANTTAFNGKQLLSGNFTNQEFQIGSSSNQTIKASIGPTQSSKIGVTRFETGSQSFTSGVVGLTIKNYNGIEDFKFDNVVISTSVGTGLGALAEEINKSADKTGVRATYDVKTTGAYAIKAGTTSQDFAINGVTIGQINYKDGDNNGQLISAINSVKDTTGVQASKDENGKLVLTSADGRGIKITGDIGVGSGILSTQKENYGRLSLVKNDGRDINVSGTGLSAIGMGAADMISQASVSLRESKGQISAANADAMGFNSYNGGGAKQILQASSISAFMSQAGSGFSAGSGFSAGSGKGYSTILSGSVQIVSSTASMSSTYVISEGSGFSAGSGNSQFAALKTSTVSAHEATAGVTTLKGAMAVMDIAETAITNLDQIRADIGSVQNQLQVTINNITVTQVNVKAAESTIRDVDFASESANFSKYNILAQSGSYAMSQANAVQQNVLKLLQ

>gi|86153031|ref|ZP_01071236.1| flagellin A [Campylobacter jejuni subsp. jejuni HB93-13]*

MGFRINTNVAALNAKANSDLNAKSLDSSLARLSSGLRINSAADDASGMAIADSLRSQANTLGQAISNGNDALGILQTADKAMDEQLKILDTIKTKATQAAQDGQSLKTRTMLQADINKLMEELDNIANTTSFNGKQLLSGGFTNQEFQIGSSSNQTVKATIGATQSSKIGVTRFETGSQSFTSGVVGLTIKNYNGIEDFKFDNVVISTSVGTGLGALAEEINKNADKTGVRATYDVKTTGAYAIKAGTTSQDFAINGVIIGKVDYKDGDNNGSLISAINAVKDTTGVQASKDENGKLVLTSADGRGIKITGDIGVGSGILSTQKENYGRLSLVKNDGRDINVSGTGLSAIGMGAADMISQASVSLRESKGQISAANADAMGFNSYNGGGAKQILQASSISAFMSQAGSGFSAGSGFSAGSGKGYSTILSGSVQIVSSTASMSSTYVISEGSGFSAGSGNSQFAALKTSTVSAHEATAGVTTLKGAMAVMDIAETAITNLDQIRADIGSIQNQVTSTINNITVTQVNVKSAESQIRDVDFASESANYSKANILAQSGSYAMAQANSSQQNVLRLLQ

>gi|87305272|ref|ZP_01087427.1| flagellin A [Campylobacter jejuni subsp. jejuni 81-176]*

MGFRINTNVAALNAKANSDLNAKSLDASLSRLSSGLRINSAADDASGMAIADSLRSQANTLGQAISNGNDALGILQTADKAMDEQLKILDTIKTKATQAAQDGQSLKTRTMLQADINKLMEELDNIANTTSFNGKQLLSGNFTNQEFQIGASSNQTVKATIGATQSSKIGVTRFETGAQSFTSGVVGLTIKNYNGIEDFKFDNVVISTSVGTGLGALAEEINKSADKTGVRATYDVKTTGVYAIKEGTTSQEFAINGVTIGKIEYKDGDGNGSLISAINAVKDTTGVQASKDENGKLVLTSADGRGIKITGDIGVGSGILANQKENYGRLSLVKNDGRDINISGTNLSAIGMGTTDMISQSSVSLRESKGQISATNADAMGFNSYKGGGKFVFTQNVSSISAFMSAQGSGFSRGSGFSVGSGKNLSVGLSQGIQIISSAASMSNTYVVSAGSGFSSGSGNSQFAALKTTAANTTDETAGVTTLKGAMAVMDIAETAITNLDQIRADIGSIQNQVTSTINNITVTQVNVKAAESQIRDVDFASESANYSKANILAQSGSYAMAQANSSQQNVLRLLQ

>gi|88596831|ref|ZP_01100068.1| flagellin subunit protein FlaA [Campylobacter jejuni subsp. jejuni 84-25]*

MGFRINTNVAALNAKANADLNSKSLDASLSRLSSGLRINSAADDASGMAIADSLRSQANTLGQAISNGNDALGILQTADKAMDEQLKILDTIKTKATQAAQDGQSLKTRTMLQADINRLMEELDNIANTTSFNGKQLLSGNFINQEFQIGASSNQTVKATIGATQSSKIGLTRFETGGRISTSGEVQFTLKNYNGIDDFQFQKVVISTSVGTGLGALADEINKNADKTGVRATFTVETRGIAAVRAGATSDTFAINGVKIGKVDYKDGDANGALVAAINSVKDTTGVEASIDANGQLLLTSREGRGIKIDGNIGGGAFINADMKENYGRLSLVKNDGKDILISGSNLSSAGFGATQFISQASVSLRESKGQIDANIADAMGFGSANKGVVLGGYSSVSAYMSSAGSGFSSGSGYSVGSGKNYSTSFANAIAISAASQLSTVYNVSAGSGFSSGSTLSQFATMKTTAFGVKDETAGVTTLKGAMAVMDIAETAITNLDQIRADIGSVQNQVTSTINNITVTQVNVKAAESQIRDVDFAAESANYSKANILAQSGSYAMAQANSVQQNVLRLLQ

>gi|9296982|sp|Q46113|FLA3_CAMJE Flagellin A *

MGFRINTNVAALNAKANADLNSKSLDASLSRLSSGLRINSAADDASGMAIKDSLRSQANTLGQAISNGNDALGILQTADKAMDEQLKILDTIKTKATQAAQDGQSLKTRTMLQADINRLMEELDNIANTTSFNGKQLLSGNFINQEFQIGASSNQTVKASIGATQSSKIGLTRFETGSRISVGGEVQFTLKNYNGIDDFKFQKVVISTSVGTGLGALADEINKNADKTGVRATFTVETRGMGAVRAGATSDDFAINGVKIGKVDYKDGDANGALVSAINSVKDTTGVEASIDENGKLLLTSREGRGIKIEGNIGRGAFINPNMLENYGRLSLVKNDGKDILISGTNLSAIGFGTGNMISQASVSLRESKGQIDANVADAMGFNSANKGNILGGYSSVSAYMSSTGSGFSSGSGFSVGSGKNYSTGFANTIAISAASQLSAVYNVSAGSGFSSGSNLSQFATMKTSAGNTLGVKDETAGVTTLKGAMAVMDIAETAITNLDQIRADIGSVQNQVTSTINNITVTQVNVKAAESQIRDVDFAAESANYSKANILAQSGSYAMAQANSVQQNVLRLLQ
