# Supplementary material for: Reducing Campylobacter jejuni Colonization of Poultry via Vaccination
Source: PLoS One. 2014 Dec 4;9(12):e114254. doi: 10.1371/journal.pone.0114254 (PMC4256221; doi:10.1371/journal.pone.0114254)
Supplement: Table S6 — Comparison of chicken colonization, sera reactivity against C. jejuni whole cell lysates, and specificity of immunoblot. (DOC) [file pone.0114254.s009.doc]

**Supplemental Table 6.** Comparison of chicken colonization, sera reactivity against *C. jejuni* whole cell lysates, and specificity of immunoblot.

| **GROUP** | **CFU/GRAM** | **ELISA Abs.** | **BLOT SPECIFICITY^a^** |
| --- | --- | --- | --- |
| **NI, No *C. jejuni*** |  |  |  |
| Bird 1 | 0 | 0.25 | Not Tested |
| Bird 2 | 0 | 0.151 | Not Tested |
| Bird 3 | 0 | 0.176 | Not Tested |
| Bird 4 | 0 | 0.170 | Not Tested |
| Bird 5 | 0 | 0.158 | Not Tested |
| Bird 6 | 0 | 0.164 | Not Tested |
| Bird 7 | 0 | 0.231 | Not Tested |
| Bird 8 | 0 | 0.218 | Not Tested |
| Bird 9 | 0 | 0.166 | Not Tested |
| Bird 10 | 0 | 0.184 | Not Tested |
| Bird 11 | 0 | 0.231 | Not Tested |
|  |  |  |  |
| **NI, + *C. jejuni*** |  |  |  |
| Bird 1 | 1.8E+08 | 0.294 | Not Tested |
| Bird 2 | 4.0E+05 | 0.403 | Not Tested |
| Bird 3 | 2.6E+09 | 1.218 | Not Tested |
| Bird 4 | 8.0E+08 | 0.805 | Not Tested |
| Bird 5 | 3.0E+08 | 0.483 | Not Tested |
| Bird 6 | 3.8E+08 | 0.741 | Not Tested |
| Bird 7 | 3.0E+07 | 0.320 | Not Tested |
| Bird 8 | 5.0E+07 | 0.609 | Not Tested |
| Bird 9 | 5.7E+07 | 0.663 | Not Tested |
| Bird 10 | 5.0E+06 | 0.819 | Not Tested |
| Bird 11 | 1.2E+05 | 0.439 | Not Tested |
| Bird 12 | 4.0E+07 | 0.582 | Not Tested |
|  |  |  |  |
| **CadF, + *C. jejuni*** |  |  |  |
| Bird 1 | 5.0E+04 | 0.721 | + |
| Bird 2 | 6.0E+04 | 1.526 | + |
| Bird 3 | 3.0E+06 | 1.844 | − |
| Bird 4 | 2.0E+06 | 1.811 | + |
| Bird 5 | 2.7E+09 | 1.727 | + |
| Bird 6 | 2.0E+04 | 0.962 | + |
| Bird 7 | 2.0E+06 | 0.569 | − |
| Bird 8 | 1.0E+06 | 1.608 | − |
| Bird 9 | 2.0E+04 | 0.360 | + |
| Bird 10 | 1.1E+06 | 1.832 | + |
| Bird 11 | 4.3E+08 | 0.745 | − |
|  |  |  |  |
| **FlaA, + *C. jejuni*** |  |  |  |
| Bird 1 | 2.1E+04 | 0.955 | + |
| Bird 2 | 1.3E+08 | 1.248 | + |
| Bird 3 | 7.0E+05 | 0.595 | − |
| Bird 4 | 3.0E+04 | 1.568 | + |
| Bird 5 | 5.0E+04 | 2.079 | + |
| Bird 6 | <10^3^ | 1.279 | + |
| Bird 7 | 6.0E+03 | 1.474 | + |
| Bird 8 | 5.0E+04 | 0.990 | + |
| Bird 9 | 1.0E+04 | 1.216 | + |
| Bird 10 | 1.5E+08 | 0.796 | + |
| Bird 11 | 2.0E+04 | 1.706 | + |
| Bird 12 | 2.0E+04 | 0.967 | + |
|  |  |  |  |
| **FlpA, + *C. jejuni*** |  |  |  |
| Bird 1 | 4.0E+04 | 0.653 | − |
| Bird 2 | 3.0E+04 | 0.826 | + |
| Bird 3 | 6.0E+05 | 0.646 | + |
| Bird 4 | 8.0E+05 | 1.304 | + |
| Bird 5 | 3.0E+05 | 0.672 | + |
| Bird 6 | <10^3^ | 0.752 | + |
| Bird 7 | 3.0E+04 | 1.278 | + |
| Bird 8 | 4.0E+03 | 0.805 | + |
| Bird 9 | 5.0E+04 | 0.836 | + |
| Bird 10 | 1.8E+05 | 0.660 | + |
|  |  |  |  |
| **Trifecta, + *C. jejuni*** |  |  |  |
| Bird 1 | 1.5E+05 | 1.683 | + |
| Bird 2 | 2.1E+04 | 0.743 | + |
| Bird 3 | 4.0E+04 | 1.608 | + |
| Bird 4 | 1.6E+04 | 0.945 | + |
| Bird 5 | 3.0E+08 | 1.789 | + |
| Bird 6 | <10^3^ | 1.584 | + |
| Bird 7 | 9.0E+04 | 1.122 | + |
| Bird 8 | <10^3^ | 1.167 | + |
| Bird 9 | 2.2E+05 | 0.762 | + |
|  |  |  |  |
| **CmeC, + *C. jejuni*** |  |  |  |
| Bird 1 | 2.0E+07 | 1.165 | Not Tested |
| Bird 2 | 1.9E+06 | 0.889 | Not Tested |
| Bird 3 | 4.0E+04 | 0.911 | Not Tested |
| Bird 4 | 1.0E+04 | 1.106 | Not Tested |
| Bird 5 | 1.1E+04 | 0.726 | Not Tested |
| Bird 6 | 3.0E+08 | 1.037 | Not Tested |
| Bird 7 | 1.2E+08 | 0.867 | Not Tested |
| Bird 8 | 1.3E+04 | 1.621 | Not Tested |
| Bird 9 | 3.0E+06 | 1.389 | Not Tested |
| Bird 10 | 1.0E+05 | 0.770 | Not Tested |
| Bird 11 | 1.2E+09 | 0.531 | Not Tested |
| Bird 12 | 1.9E+07 | 0.694 | Not Tested |

^a^Blot specificity defined as reactivity against the peptide of interest in a *C. jejuni* whole cell lysate, based on Supplemental Figure 1.
