# Supplementary material for: Reducing Campylobacter jejuni Colonization of Poultry via Vaccination
Source: PLoS One. 2014 Dec 4;9(12):e114254. doi: 10.1371/journal.pone.0114254 (PMC4256221; doi:10.1371/journal.pone.0114254)
Supplement: Materials and Methods S1 — Vaccination of chickens (pilot experiment). (DOC) [file pone.0114254.s010.doc]

**Supplemental Materials and Methods:**

*Vaccination of chickens (pilot experiment)*

Sixty-seven chicks were subdivided into eight groups, with eight or nine chicks per group. At 6 days of age, the chicks were immunized with 72 µg of the GST-tagged 90mer peptides GST-FlaA, GST-FlpA, GST-CjaA, GST-CmeC, and GST-trifecta (see Table 3), or 14.5 µg of GST-CadF. At 16 days of age, a booster injection was given with 240 µg of full-length CadF-His and FlpA-His, or 240 µg 90mer GST-FlaA, GST-CjaA, GST-CmeC, and GST-trifecta. The peptides (antigens) were emulsified in Montanide ISA 70 VG (Seppic, Paris, France) at a ratio of 30% antigen and 70% Montanide 70 VG. For the primary and booster injections, 200 µL of peptide/adjuvant mixture was injected into the left and right breast muscle. Two groups were not immunized, with one group serving as the negative control uninfected chicks, and the other as the positive control chicks infected with *C. jejuni* but not vaccinated. The pilot experiment was performed at Washington State University in a different facility than that was used for the experiment in the manuscript.

The *C. jejuni* challenge experiments and sera analysis were performed as described in the Materials and Methods of the manuscript.
